# Nonequilibrium Arrhythmic States and Transitions in a Mathematical Model for Diffuse Fibrosis in Human Cardiac Tissue

**DOI:** 10.1371/journal.pone.0045040

**Published:** 2012-10-08

**Authors:** Rupamanjari Majumder, Alok Ranjan Nayak, Rahul Pandit

**Affiliations:** 1 Centre for Condensed Matter Theory, Department of Physics, Indian Institute of Science, Bangalore, India; 2 Jawaharlal Nehru Centre for Advanced Scientific Research, Bangalore, India; Temple University, United States of America

## Abstract

We present a comprehensive numerical study of spiral-and scroll-wave dynamics in a state-of-the-art mathematical model for human ventricular tissue with fiber rotation, transmural heterogeneity, myocytes, and fibroblasts. Our mathematical model introduces fibroblasts randomly, to mimic diffuse fibrosis, in the ten Tusscher-Noble-Noble-Panfilov (TNNP) model for human ventricular tissue; the passive fibroblasts in our model do not exhibit an action potential in the absence of coupling with myocytes; and we allow for a coupling between nearby myocytes and fibroblasts. Our study of a single myocyte-fibroblast (MF) composite, with a single myocyte coupled to 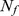 fibroblasts via a gap-junctional conductance 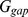, reveals five qualitatively different responses for this composite. Our investigations of two-dimensional domains with a random distribution of fibroblasts in a myocyte background reveal that, as the percentage 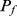 of fibroblasts increases, the conduction velocity of a plane wave decreases until there is conduction failure. If we consider spiral-wave dynamics in such a medium we find, in two dimensions, a variety of nonequilibrium states, temporally periodic, quasiperiodic, chaotic, and quiescent, and an intricate sequence of transitions between them; we also study the analogous sequence of transitions for three-dimensional scroll waves in a three-dimensional version of our mathematical model that includes both fiber rotation and transmural heterogeneity. We thus elucidate random-fibrosis-induced nonequilibrium transitions, which lead to conduction block for spiral waves in two dimensions and scroll waves in three dimensions. We explore possible experimental implications of our mathematical and numerical studies for plane-, spiral-, and scroll-wave dynamics in cardiac tissue with fibrosis.

## Introduction

Extra-cellular-matrix (ECM) materials constitute about 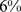 of the volume of human cardiac tissue in an average, healthy heart [1]. These include fibroblasts, non-excitable collagen, and elastin fibrils, which fill the subepicardial space between the epicardium and myocardium [2] and bridge the gaps between myocardial tissue layers. The major component of the ECM are fibroblast cells that produce interstitial collagen, of types I, III, IV, and VI [3]. These contribute to myocardial structure, cardiac development, cell-signaling, and electro-mechanical functions in myocardial tissue. In mammalian cardiac tissue, fibroblast cells show an intimate spatial interrelation with every myocyte that borders one or more fibroblasts [4]. In tissue containing both myocytes and fibroblasts, it has been assumed traditionally that gap-junctional couplings exist exclusively between myocytes; but recent experimental studies have shown that there is a functional, heterogeneous, myocyte-fibroblast coupling [3], [4].

Computer simulations of electrical-wave propagation in mathematical models for cardiac tissue have been used to investigate the interplay of spiral and scroll waves with conduction and other inhomogeneities [1], [5]–[21]. Some of these studies [5], [6], [14]–[18] concentrate on the interaction of a spiral or scroll wave with a localized inhomogeneity; others are devoted to investigations of the effects of a large number of randomly-distributed, point-type inexcitable obstacles on such waves [1], [7]–[13]. We concentrate on the latter types of studies here because they have been designed to mimic arrays of fibrotic strands or diffuse fibrosis in cardiac tissue. By using a simple model for cardiac tissue with many inexcitable obstacles, Pertsov [7] has shown that such obstacles can influence the rotation of spiral waves and lead to anisotropies in propagation. Turner, *et al.* [8] have studied the effects of fibrosis in the Priebe-Beukelmann model. Spach, *et al.* [9] have used the Nygren model for human atrial tissue and mimicked the effects of diffuse fibrosis by removing lateral gap junctions; they find that with such heterogeneity in intercellular couplings, there is a tendency for partial wave block and re-entry. Kuijpers, *et al.* [10] have used the Courtemanche model for human atrial tissue and heterogeneous uncoupling to model diffuse fibrosis. These studies indicate that fibrosis can increase vulnerability to re-entry; however, they have not explored in detail the effects of fibrosis on the dynamics of spiral and scroll waves in these models. Such an exploration has been initiated by Panfilov [11] and ten Tusscher, *et al.* [1], [12], [13], who investigate the effects of diffuse fibrosis on the propagation of electrical waves of activation and arrhythmogenesis in both two-variable and detailed ionic mathematical models for human ventricular tissue; they model fibrosis as non-conducting inhomogeneities that are distributed randomly in their simulation domain. They show that, as the concentration of such inhomogeneities increases, 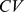 decreases for plane-wave propagation, the wave fronts become jagged, and there is an increase in the tendency for the formation and break up of spiral waves; at sufficiently large densities of these inhomogeneities, they find that complete conduction blockage occurs.

McDowell, *et al.* [22] have used a three-dimensional computational model, based on MRI data, of chronically infarcted rabbit ventricles to characterize arrhythmogenesis because of myofibroblast infiltration as a function of myofibroblast density; this study includes periinfarct zones (PZ), ionic-current remodeling therein, and different degrees of myofibroblast infiltration. Their work shows that, at low densities, myofibroblasts do not alter the propensity for arrhythmia; at intermediate densities, myofibroblasts cause AP shortening and thus increase this propensity; at high densities, these myofibroblasts protect against arrhythmia by causing resting depolarization and blocking propagation.

We present a major extension of the work of ten Tusscher, *et al.* [1], [12], [13] on diffuse fibrosis in mathematical models for cardiac tissue by introducing fibroblasts randomly in the state-of-the-art TNNP model [16], [23] for human ventricular tissue; the fibroblasts are passive, insofar as they do not exhibit an action potential in the absence of coupling with myocytes. Our model for the fibroblasts is much more realistic than the one used by ten Tusscher, *et al.* [1], [12], [13]; in particular, we use the fibroblast model of MacCannell, *et al.* [24], [25]; we also allow coupling between nearby myocytes and fibroblasts. The parameters in this model cannot be determined precisely from experiments [4], [25] so it is important to explore a wide, but biophysically relevant, range of parameters. Our *in silico* study is well suited for such an exploration so it is very effective in complementing experimental studies of electrical-wave propagation in fibrotic cardiac tissue.

We begin with an overview of our principal results before we present the details of our study. We first study a single myocyte-fibroblast (MF) composite in which a single myocyte is coupled to 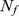 fibroblasts via a gap-junctional conductance 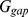. We study two cases, namely, moderate and strong coupling between fibroblasts and myocytes; for each one of these cases, we consider three parameter sets [23] for the myocytes that are suitable for epicardial, mid-myocardial, and endocardial layers of the heart wall; experiments suggest that 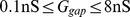 [26], [27]; thus, we consider 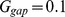 nS, 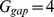 nS, and 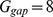 nS to be the weak-, moderate- and strong-coupling cases, respectively. We excite each such MF composite via an electrical stimulus and then record its responses for different values of 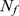 and 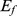. In the moderate-coupling case (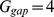 nS), the electrical load of the fibroblasts on the myocyte is not significant, except in a very narrow range of parameters. However, in the strong-coupling case (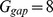 nS), for different ranges of the parameters 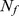 and 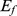, we observe five qualitatively different responses for the MF composite; we call them **R1–R5**. In **R1** the MF composite responds essentially like an uncoupled myocyte. In régime **R2**, the MF composite produces a secondary AP, after the first one that is generated by the external stimulus. In **R3**, this composite displays autorhythmicity, i.e., it fires a train of APs, after the first external stimulus, and without the application of subsequent stimuli; each AP in this autorhythmic train differs from the normal AP of an uncoupled mycocyte. In régime **R4**, the MF composite displays an oscillatory state in which the initial AP response to the external stimulus is followed by oscillations of the membrane potential about a mean value without the application of any other external stimulus. In régime **R5**, the MF composite produces a single AP under the influence of the external stimulus; after that it does not return to the resting state but to another time-independent state in which it is non-excitable.

We then study propagation of plane waves in a 2D simulation domain with TNNP-type [16], [23] myocytes (M) or fibroblasts (F) of the type described in Ref. [24]; M and F are distributed randomly through the simulation domain; and there are diffusive couplings between nearest-neighbor cells. We investigate plane-wave propagation through both mural slices, with epicardial parameters, and transmural slices, consisting of epicardial, mid-myocardial, and endocardial regions, in moderate- and strong-coupling cases, i.e., with myocyte-to-fibroblast diffusion constants of 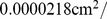
ms and 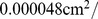
ms, respectively. We obtain stability diagrams for both these cases in the 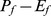 parameter space. In the moderate-coupling case, this stability diagram is simple: for low values of 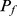 the plane wave leaves the system, which returns to an excitable state; for large values of 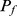 the plane wave is annihilated by target waves and the medium is left in a state that is weakly excitable or not excitable at all. In the strong-coupling case the stability diagram is very rich; it contains the spatiotemporal analogs of the régimes **R1–R5** mentioned above for an isolated MF composite.

The last part of our study examines the effect of diffuse fibrosis on spiral-wave dynamics in 2D and scroll-wave dynamics in 3D with myocytes and fibroblasts distributed randomly as above; we concentrate on the strong-coupling case here. At low values of 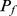, we find that single, rotating spiral and scroll waves have slightly corrugated wave fronts, but they propagate much as they do in the absence of fibroblasts. For large values of 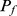, we find that such spiral and scroll waves do not propagate through the simulation domain and are either (a) annihilated by spontaneously generated target waves or (b) absorbed at the boundaries. This crossover from a state with propagating waves and electrical activity to a state with no electrical activity occurs via a sequence of nonequilibrium transitions; the precise sequence depends on the initial conditions and the realization of the disordered array of M and F cells.

Given the spatial and temporal resolution we have been able to achieve in 2D, we find the following rough sequence of states: at low 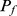 we begin with a state with a single spiral rotating periodically (**SRSP**); as 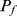 increases this gives way to a state with a single spiral rotating quasi-periodically (**SRSQ**); as 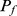 increases we obtain a state with multiple spirals that rotate periodically (**MRSP**); this then gives way to a state with a multiple spirals that rotate quasi-periodically (**MRSQ**), which is followed by a spiral-turbulence (**ST**) state and, eventually, by the absorption state (**SA**). In 3D, the analogous sequence of states, which we have been able to resolve, is the following: at low 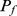 we begin with a state with a single rotating scroll wave (**SRS**); as 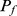 increases this gives way to a state with multiple rotating scroll waves (**MRS**); this then gives way to the absorption state (**SA**).

## Materials and Methods

The first system we study is a single myocyte-fibroblast (MF) composite in which a single myocyte is coupled to 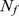 fibroblasts via a gap-junctional conductance 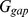; we consider the range 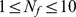. We then carry out studies in 2D and 3D simulation domains in which myocytes M or fibroblasts F are distributed randomly through the simulation domain; we include diffusive couplings between these. The precise ionic models and the diffusive couplings are described in detail below.

In 2D we use a square simulation domain (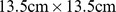), when we consider a mural slice, and a rectangular domain (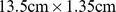), when we study a transmural slice. This 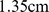 thick transmural slice is further divided into three parallel strips of width 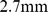 for the epicardium, 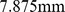 for the mid-myocardium, and 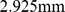 for the endocardium. For the mural slice, we choose parameters for epicardial-type myocytes, whereas, for the transmural slice, we use parameters for epicardial, mid-myocardial, and endocardial myocytes in the appropriate strips. In 3D our simulation domain is a rectangular parallelepiped of physical size 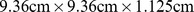. Our spatial grid uses 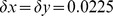 cm in 2D and 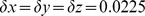 cm in 3D; and our time-marching scheme has a time step 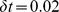 ms.

The actual thickness of the human left ventricular wall varies between 1 and 2 cm (see Ref. [28]); we have chosen a thickness of 1.35 cm because it is well within this range. For our 3D simulations, we have reduced this thickness to 1.125 cm for the following reasons: (a) we have checked, in a few representative cases, that the principal results of our study do not change qualitatively if we reduce the thickness of the domain by a few millimeters; (b) our 3D simulations are computationally very expensive because we need to run the simulations for long time and store several intermediate configurations to distinguish the states SRS, MRS, and SA; (c) a wall thickness of 1.125 cm also lies within the acceptable range of thicknesses for the left ventricular wall. The precise ratios of the thicknesses of the three layers of the heart wall are not known. However, a rough estimate (see Ref. [28]) indicates that the epicardium is, on average, 2–3 mm thick, the mid-myocardium is the thickest zone, and the endocardium has a highly non-uniform thickness; the values we have chosen for the thicknesses of the epicardial, mid-myocardial, and endocardial slices in our simulations are commensurate with these rough estimates and observations (see Ref. [17]).

For the ionic activity of the myocytes, we adopt the state-of-the-art TNNP model [23] for human cardiac tissue, which is the following reaction-diffusion equation for the transmembrane potential 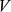:
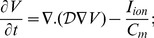
(1)here 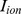, the total ionic current, is expressed as a sum of the following six major and six minor ionic currents:
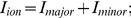
(2)

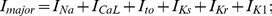


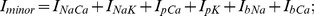
here 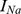 is the fast inward 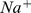 current, 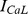 the 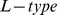 slow-inward 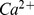 current, 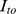 the transient outward current, 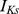 the slow, delayed-rectifier current, 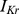 the rapid, delayed-rectifier current, 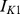 the inward rectifier 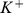 current, 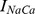 the 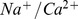 exchanger current, 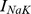 the 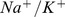 pump current, 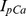 the plateau 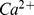 current, 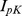 the plateau 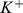 currents, 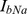 the background 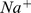 current, and 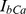 the background 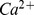 current. The time 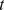 is measured in milliseconds, voltage 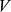 in millivolts, conductances (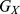) in nanoSiemens per picofarad (nS/pF), the intracellular and extracellular ionic concentrations (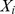, 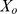) in millimoles per liter (mM/L) and current densities, per unit capacitance, 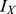 in picoamperes per picofarad (pA/pF), as used in second-generation models (see, e.g., Refs. [23], [29]–[31]). For a detailed list of the parameters of this model and the equations that govern the spatiotemporal behaviors of the transmembrane potential and currents, we refer the reader to Refs. [16], [23].

For the fibroblasts, we use the model of MacCannell, *et al.* [24]; i.e., we treat the fibroblasts as passive circuit elements that couple with the myocyte in the MF composite; and the fibroblast ionic current 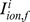 is
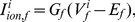
(3)where 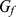, 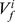 and 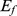, are, respectively, the conductance, transmembrane potential, and the resting membrane potential for the fibroblast.

We incorporate muscle-fiber anisotropy in both 2D and 3D simulations, as in Refs. [32], [33]; we account for diffusive couplings between nearest-neighbor myocytes, nearest-neighbor fibroblasts, next-nearest neighbor myocytes, next-nearest-neighbor fibroblasts and nearest-neighbor myocyte-fibroblast pairs. We use two diffusion tensors, namely, 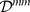 and 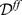, for myocyte-myocyte (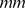) and fibroblast-fibroblast (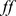) diffusive couplings. The diffusion tensors 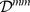 and 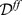 have the form used in Refs. [32], [34]; we give this form below for a diffusion tensor that is denoted generically by 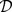 and has, in three dimensions, the components shown hereunder:
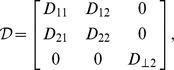
where
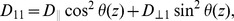


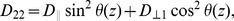


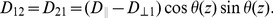
(4)For myocyte-myocyte couplings, we use 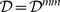; 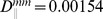 cm
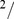
ms is the diffusivity for propagation through myocytes, parallel to the fiber axis, 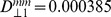 cm
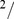
ms the diffusivity through myocytes, perpendicular to this axis but in the same plane, and 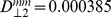 cm
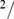
ms the diffusivity through myocytes, perpendicular to the fiber axis but out of the plane, i.e., in the transmural direction. The twist angle along the transmural direction is 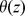. It is related to the rate of fiber rotation 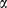 via 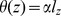, where 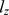 is the thickness of the tissue as measured from the bottom of the endocardium, along the 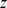 axis. The total fiber rotation (FR) across the slab is taken to be 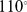, which is the typical value for the human ventricular wall. For fibroblast-fibroblast couplings 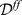 also has the same form as 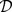; we choose 
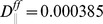
 cm
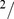
ms and 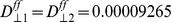 cm
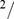
ms because we expect 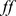 diffusive couplings to be much smaller than their 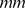 counterparts. In 2D simulations, we use only the components 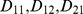, and 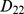 for a mural slice in the 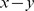 plane; for a transmural slice in the 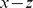 plane we retain only the components 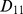 and 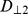.

We turn now to 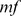 and 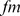 diffusive couplings; the magnitudes of these are not known well experimentally, nor has the role of fiber orientation been investigated in this context. Therefore, in the interests of a parsimonious description, we neglect fiber orientation in the 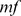 and 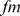 diffusive couplings 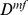 and 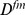, respectively, and treat them as scalars. In keeping with the idea that the interaction between a myocyte and a fibroblast should be weaker than that between two myocytes, but stronger than that between two fibroblasts, we use the following illustrative values: (a) for the strong-coupling case 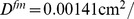
ms and 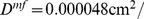
ms; and (b) for the moderate-coupling case 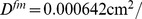
ms and 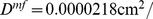
ms; in both these cases 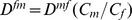, where the total cellular capacitances for myocytes and fibroblasts are 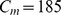 pF and 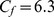 pF, respectively [24]. Note that, in our 2D and 3D models, there is no on-site coupling between myocytes and fibroblasts; this has been translated into diffusive couplings between such cells if they are at nearest-neighbor sites in our simulation domains.

**Figure 1:**
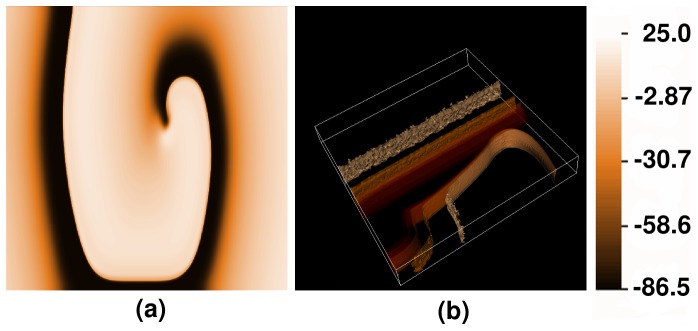
The initial configurations for the spiral and scroll waves in our 2D and 3D simulations (see text).

We generate the initial condition for our studies by using the following protocols: We begin with only myocytes on all sites of our 2D simulation domain. For plane-wave-propagation studies, we apply a stimulus, of amplitude 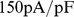 for 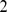 ms, along one edge of the simulation domain. For our studies of spiral-wave dynamics, we obtain a spiral wave in the 2D domain by using the method proposed by Shajahan, *et al.* [16]. In our 3D scroll-wave studies, we begin with an initial scroll wave that consists of our initial, 2D spiral waves stacked one on top of the other; thus, we begin with a simple scroll wave with a straight filament as in Ref. [17]. Pseudocolor plots of 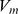 for these spiral and scroll waves, which we use as initial conditions in our subsequent studies, are given in Figs. 1 (a) and (b), respectively.

**Figure 2:**
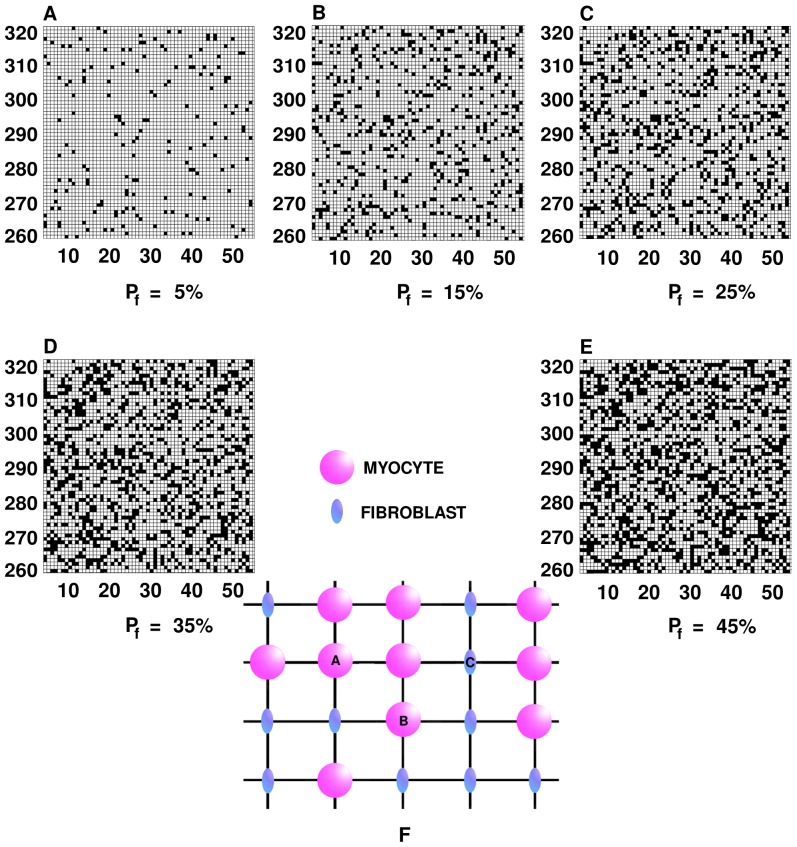
Spatial distributions of myocytes and fibroblasts in our simulation domain. Illustrative plots of the distributions of fibroblasts and myocytes, on a representative 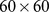 part of our simulation domain in two dimensions (2D). Fibroblasts (filled black squares) and myocytes (unfilled white squares) are distributed randomly in our simulation domain so that a given grid point contains either a myocyte or a fibroblast, but not both; the percentage of fibroblasts 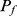 is (A) 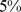, (B) 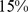, (C) 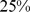, (D) 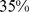, and (E) 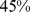. In (F) we show an illustrative arrangement of myocytes (pink circles) and fibroblasts (blue ellipses); for such an arrangement the diffusion terms are as given in Eqs. 6–8.

For every value of 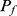, we generate a random array of myocytes and fibroblasts in our 2D and 3D simulation domains as follows by using a random-number generator to assign F or M to a site such that the percentage of F sites is 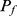; illustrative arrays of F and M are given in Fig. 2 (a)–(e). This distribution of F and M cells is held fixed throughout the subsequent spatiotemporal evolution of the initial spiral and scroll waves described above; in the language of condensed-matter physics, a static configuration of F and M is an example of quenched disorder [35]–[37]. At the initial time, the fibroblast transmembrane potential 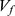 is set equal to its resting value 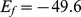 mV [24].

The temporal evolution of the transmembrane potential 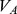 of the cell at site 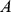 in the lattice is governed by
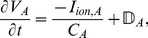
(5)where 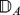 indicates the diffusion term. This can be written most easily in discrete form and it depends on (a) whether the cell at site 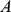 is M or F and (b) whether the cell on the neighboring site is of type M or F. We illustrate the form of the diffusion term 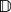 for a two-dimensional mural slice for three representative sites 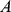, 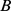, and 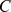 in Fig. 2(f), which have M, M, and F cells, respectively:
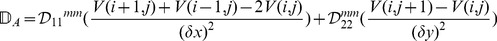
(6)

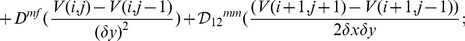


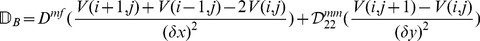
(7)

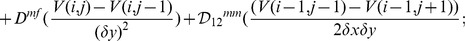


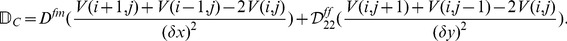
(8)


In our studies with the MF composite, we apply a stimulus current of 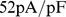 for 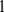 ms to the composite and allow the system to evolve in time. We record the membrane potential of the central myocyte in the MF composite and plot it as a function of time for different values of 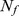 and 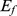.

For our measurements of 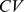 and 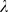, we prepare the 2D simulation domain as discussed above and initiate a plane wave by applying a stimulus of amplitude 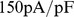 for 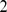 ms along the left edge (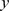 axis) of the domain. We record the time series of 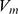 at four representative points of the domain. For studies on the mural slice, these points are (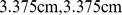), (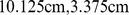), (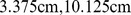) and (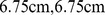); for studies on the transmural slice, these points are (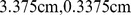), (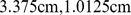), (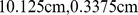), (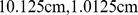) and (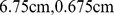). From these time-series data, we obtain the times 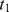 and 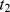 at which the upstroke of the action potential (AP) is initiated at pairs of points that are separated by 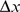 along the axis parallel to the direction of propagation of the wave; 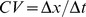, where 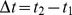; the wavelength 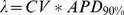, where 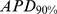 is the action-potential duration at 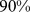 repolarization; we obtain average values for 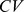 and 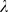 over the four points mentioned above.

## Results

**Figure 3:**
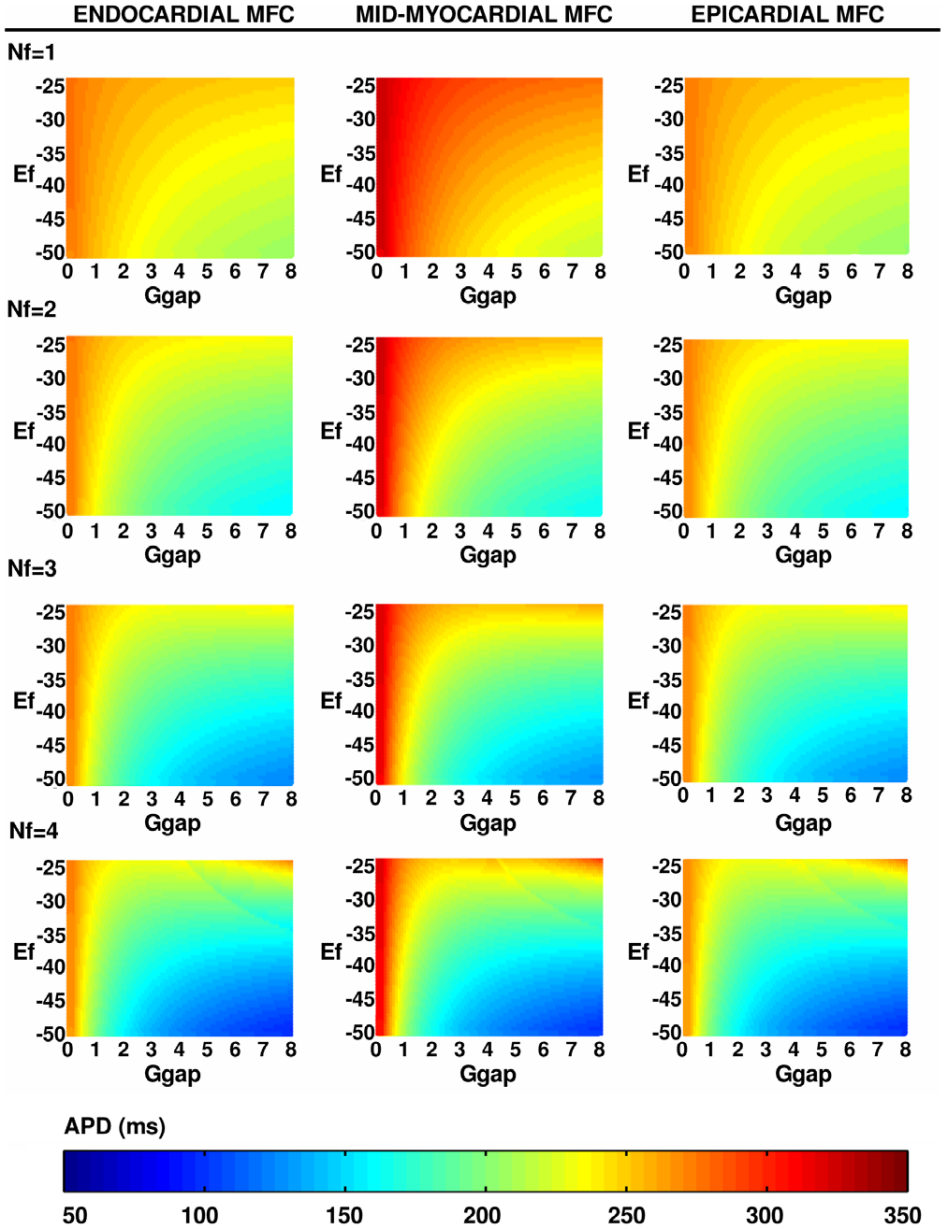
Dependence of action potential duration (APD) of an MF composite on 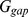 and 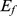. Illustrative pseudocolor plots of the APD for MFs, with epicardial, mid-myocardial, and endocardial myocytes, as functions of the gap-junctional conductance 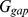 and the resting membrane potential of fibroblasts 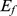, for the number of coupled fibroblasts 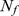 = 1, 2, 3, and 4.

In this Section we present the results of our computational studies. We begin with our investigation of MF composites and discuss how their action potential is influenced by the number of fibroblasts 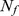, their resting membrane potential 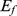, the gap-junctional coupling 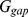, and the myocyte parameters, which distinguish myocytes from the epicardium, the mid-myocardium, and the endocardium. We then explore the propagation of plane waves of electrical activation through 2D simulation domains with randomly distributed myocytes and fibroblasts such that the percentage of fibroblasts is 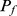; we consider propagation through both mural and transmural slices. Next we consider spiral-wave dynamics in 2D and 3D simulation domains with 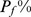 fibroblasts. The action potential durations (APDs) are different, for uncoupled myocytes from the endocardium, the mid-myocardium and the epicardium. The APD of the myocyte-fibroblast composite (MF) is also different for the three types of myocytes. However, the APD of an MF depends not only on the type of myocyte but also on the values of the gap-junctional conductance 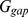, the resting membrane potential of fibroblasts (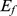), and the number of fibroblasts coupled to a myocyte (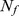). Fig. 3 shows pseudocolor plots of the APD for MFs, with epicardial, mid-myocardial, and endocardial myocytes, as functions of 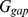 and 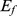, for 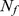 = 1, 2, 3, and 4. In our studies, 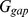 is moderate (4 nS) or strong (8 nS), so the influence of the gap-junctional coupling is quite significant. When 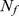 = 1, the differences between the APDs, for the three types of MFs, is considerable, for all values of 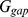 and 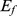; as 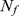 increases, this difference between the APDs is significant only at low values of 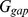; in particular, for 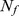 = 4 and 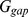 = 8 nS, the distinction between these APDs is almost negligible. However, if 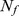 = 4 and 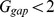, this distinction is quite prominent at all the values of 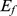 that we have considered. For studies on transmural heterogeneity in mouse tissue, please refer to [38], [39].

### MF Composite

**Figure 4:**
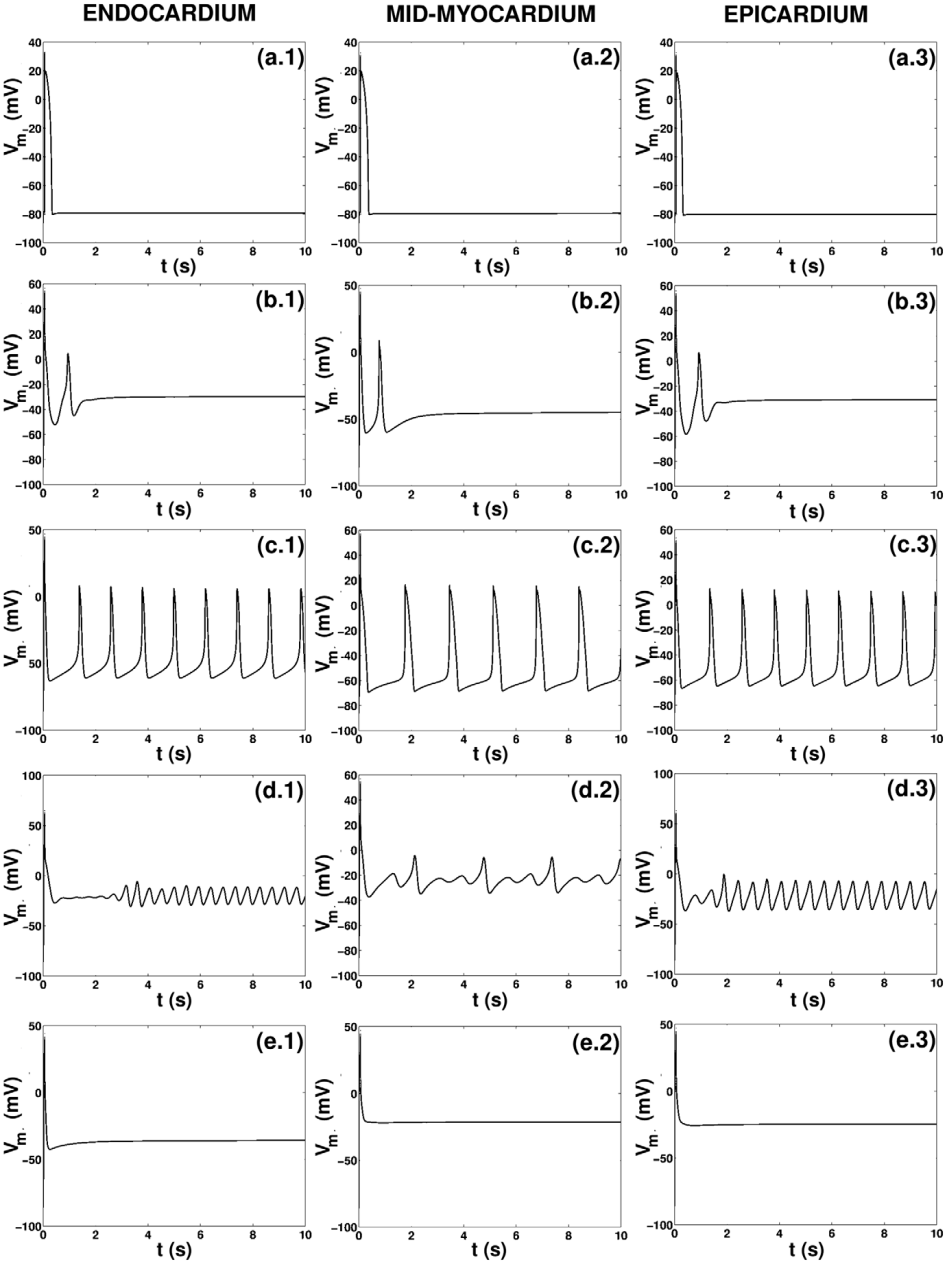
Representative time series of the transmembrane potential recorded from an MF composite. Plots of the transmembrane potential 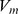 of the myocyte in the myocyte-fibroblast (MF) composite versus time 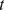 for the strong-coupling case 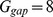 nS showing the following: responses of type **R1** for 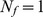, 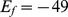 mV, and (a.1) epicardial myocytes, (a.2) mid-myocardial myocytes, and (a.3) endocardial myocytes; responses of type **R2** for (b.1) epicardial myocytes and 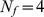, and 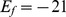 mV, (b.2) mid-myocardial myocytes and 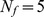, and 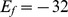 mV, and (b.3) endocardial myocytes and 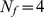, and 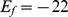 mV; autorhythmic responses of type **R3** for (c.1) epicardial myocytes and 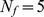, and 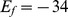 mV, (c.2) mid-myocardial myocytes and 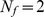, and 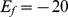 mV, and (c.3) endocardial myocytes and 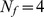, and 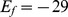 mV; oscillatory responses of type **R4** for (d.1) epicardial myocytes and 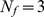, and 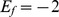 mV, (d.2) mid-myocardial myocytes and 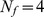, and 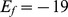 mV, and (d.3) endocardial myocytes and 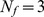, and 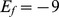 mV; responses of type **R5** for (e.1) epicardial myocytes and 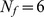, and 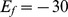 mV, (e.2) mid-myocardial myocytes and 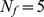, and 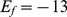 mV, and (e.3) endocardial myocytes and 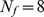, and 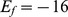 mV.

The response of the myocyte-fibroblast (MF) composite depends on 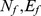, and 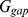 and the properties of the myocyte. In both moderate- and strong-coupling cases, with 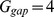 nS and 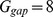 nS, respectively, if 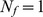 the MF composite produces a single action potential on the application of an external stimulus and then returns to the normal resting membrane potential for myocytes (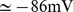); we designate this as a response of type **R1**; and we illustrate this, for the case 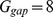 nS, by plots of 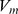 versus time 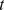 in Figs. 4 (a.1), (a.2), and (a.3) for epicardial, mid-myocardial, and endocardial myocytes, respectively. Four other types of responses are possible and are listed below and portrayed in Fig. 4, for the case 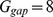 nS: **R2**: In this case there is a secondary AP, after the first one generated by an external stimulus; the MF composite then returns to the resting state as in **R1** (Figs. 4 (b.1), (b.2), and (b.3) for epicardial, mid-myocardial, and endocardial myocytes, respectively). **R3**: Here the MF composite is autorhythmic, i.e., it produces a sequence of APs, after the first external stimulus; each AP in this autorhythmic sequence differs from the normal AP of an uncoupled mycocyte (Figs. 4 (c.1), (c.2), and (c.3) for epicardial, mid-myocardial, and endocardial myocytes, respectively). **R4**: The MF composite can display an oscillatory response in which the initial, stimulus-induced AP is followed by oscillations of 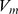, about a mean value, without the application of any other external stimulus (Figs. 4 (d.1), (d.2), and (d.3) for epicardial, mid-myocardial, and endocardial myocytes, respectively). **R5**: The MF composite produces a single AP because of an external stimulus; after that it does not return to the normal resting state but to another time-independent state in which it is non-excitable (Figs. 4 (e.1), (e.2), and (e.3) for epicardial, mid-myocardial, and endocardial myocytes, respectively).

**Figure 5:**
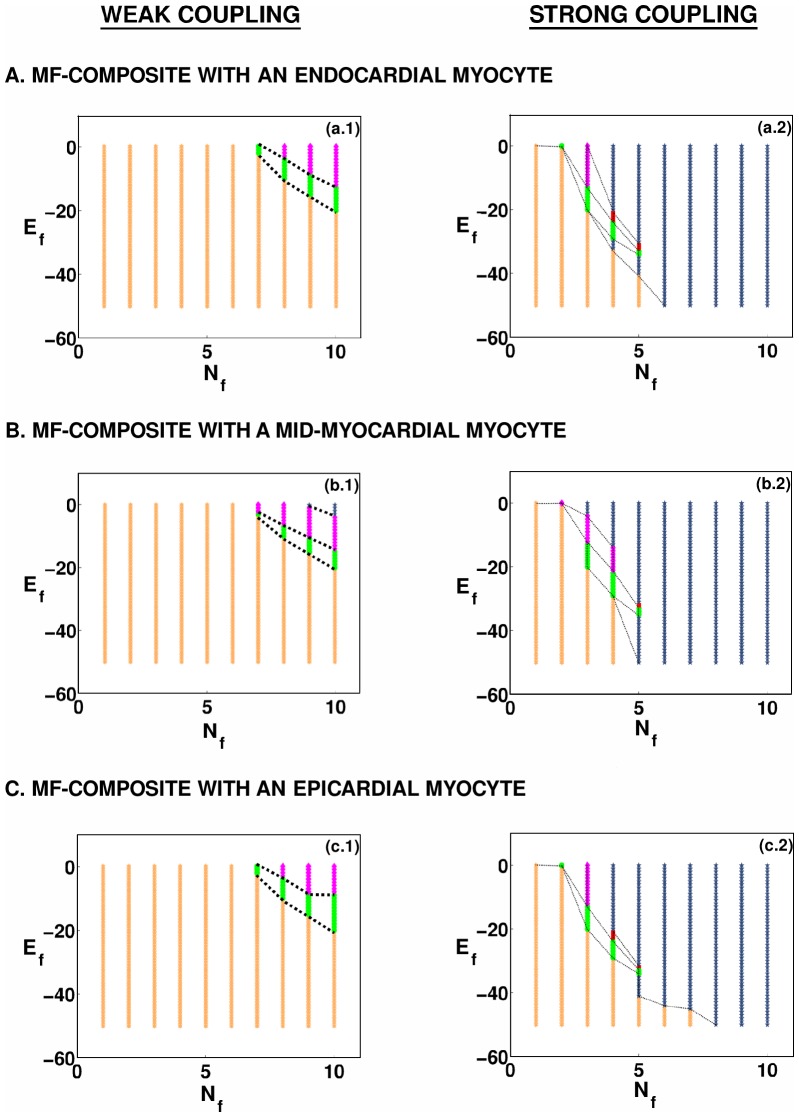
Stability diagrams in the 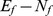 parameter space for the responses of an MF composite. The regions in which the MF composite shows responses of types **R1–R5** are shown in (a.1), (a.2), (b.1), (b.2), (c.1), and (c.2). For the moderate-coupling case, 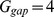 nS, (a.1), (b.1), and (c.1) show the stability diagrams for, respectively, endocardial, mid-myocardial, and epicardial myocytes in the MF composite; their analogs for the strong-coupling case, 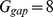 nS, are given in (a.2), (b.2), and (c.2); the régimes **R1**, **R2**, **R3**, **R4**, and **R5** are denoted, respectively, by yellow hexagrams, red squares, green circles, pink diamonds, and blue pentagrams.

The regions in which our MF composite displays responses of types **R1–R5** are shown, for illustrative parameter values, in the 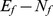 plane in Figs. 5 (a.1), (a.2), (b.1), (b.2), (c.1) and (c.2). For the moderate-coupling case, 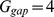 nS, Figs. 5 (a.1), (b.1), and (c.1) show the stability diagrams for, respectively, endocardial, mid-myocardial, and epicardial myocytes in the MF composite; their analogs for the strong-coupling case, 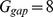 nS, are given in Figs. 5 (a.2), (b.2), and (c.2); here régimes **R1**, **R2**, **R3**, **R4**, and **R5** are denoted, respectively, by yellow hexagrams, red squares, green circles, pink diamonds, and blue pentagrams. All these régimes appear in stability diagrams for the moderate- and strong-coupling cases; but régimes **R3** and **R4** occupy very small areas especially in the moderate-coupling case; and régime **R5**, which occupies a significant fraction of the stability diagram in the strong-coupling cases, occurs in a narrow parameter range in the case of moderate coupling, but only when we consider MF composites with mid-myocardial myocytes.

### Plane-wave propagation in a 2D domain

We now investigate the propagation of plane waves of electrical activation through a 2D simulation domain of the type we have described above. In this domain, we distribute myocytes and fibroblasts randomly such that the percentage of fibroblasts is 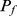; we consider propagation through both mural and transmural slices. In addition to 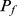, the other important parameters in this part of our study are 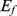 and the components of the diffusion tensors, i.e., 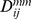, 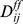, and 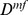 and 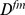 (see Eqs. 4). Recall that, in our 2D and 3D models, there is no on-site coupling between myocytes and fibroblasts; but we have diffusive couplings between such cells if they are at nearest-neighbor sites in our simulation domains; here 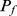 plays a rôle similar to that of 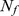, in our studies of MF composites. We show below that the temporal responses, of types **R1–R5**, for MF-composites, have spatiotemporal analogs when we consider plane-wave propagation through our 2D simulation domain; we denote these analogs by the same symbols, namely, **R1–R5**, because the spatiotemporal evolution of the plane waves in these stability régimes can be rationalized, qualitatively, in terms of the responses of MF composites that we have discussed above.

**Figure 6:**
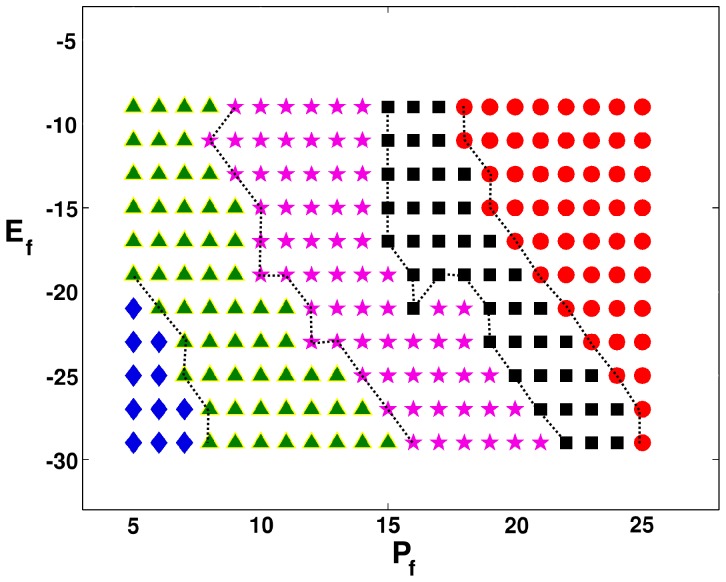
Stability diagram in the 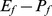 plane for plane-wave propagation through a mural slice of our 2D simulation domain with a random distribution of myocytes and fibroblasts. The stability diagram shows the regions with spatiotemporal behaviors **R1–R5** in the strong-coupling case (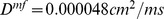); the regions **R1**, **R2**, **R3**, **R4**, and **R5** are denoted, respectively, by blue diamonds, green triangles, pink pentagrams, black squares, and red circles; the spatiotemporal evolution of plane waves in these regions is described in the text.

We first consider plane-wave propagation through a mural slice. We find the five qualitatively different spatiotemporal behaviors **R1–R5**. In the régime **R1**, which occurs both in moderate- and strong-coupling cases, the plane wave propagates smoothly through the simulation domain but with a slightly corrugated wave front. In the régime **R2**, small clusters of fibroblasts can form around some sites with myocytes; these clusters have the capacity to generate one subsidiary action potential (cf. the response **R2** of an MF composite), before returning to a resting potential that is above the resting potential of the myocytes; because of this subsidiary action potential, target waves are generated by the fibroblast clusters and a plane wave, which tries to propagate through the simulation domain, is annihilated by these target waves, so the whole domain returns to a potential that is above the normal resting membrane potential of myocytes, but below their threshold potential; **R2** is absent in the moderate-coupling case, in the parameter régimes that we have explored. The parameter régime **R3** is characterized by autorhythmicity; the fibroblast clusters about some myocyte now develop the ability to sustain rhythms of their own (cf. the response **R3** of the MF composite); here too the initial plane wave is annihilated by the target waves that are generated by the autorhythmic fibroblast clusters; however, unlike in the case **R2**, the activity of our medium does not stop here; after a considerable length of time, the autorhythmic fibroblast clusters generate sustained beats of their own; beats from fibroblast clusters of different sizes, which are in different parts of the medium, are incoherent; **R3** is absent in the moderate-coupling case, in the parameter régimes that we have explored. In the oscillatory regime régime **R4** (cf. the response **R4** of the MF composite), the fibroblast clusters produce an initial target wave that annihilates the plane wave; this is followed by temporal oscillations, about some mean potential, of the local membrane potential; **R4** is absent in the moderate-coupling case, in the parameter régimes that we have explored. In régime **R5** the initial plane wave is terminated by collisions with numerous target waves, which are generated by the fibroblast clusters that are distributed randomly throughout the medium; once the plane wave is removed, the medium moves into a quiescent state with a membrane potential that lies above the excitation-threshold potential for an uncoupled myocyte; no further excitation is possible; **R5** is absent in the moderate-coupling case, in the parameter régimes that we have explored. The stability diagram, which shows the regions with spatiotemporal behaviors **R1–R5** in the strong-coupling case, is given in Fig. 6; regions **R1**, **R2**, **R3**, **R4**, and **R5** are denoted, respectively, by blue diamonds, green triangles, pink pentagrams, black squares, and red circles.

**Figure 7:**
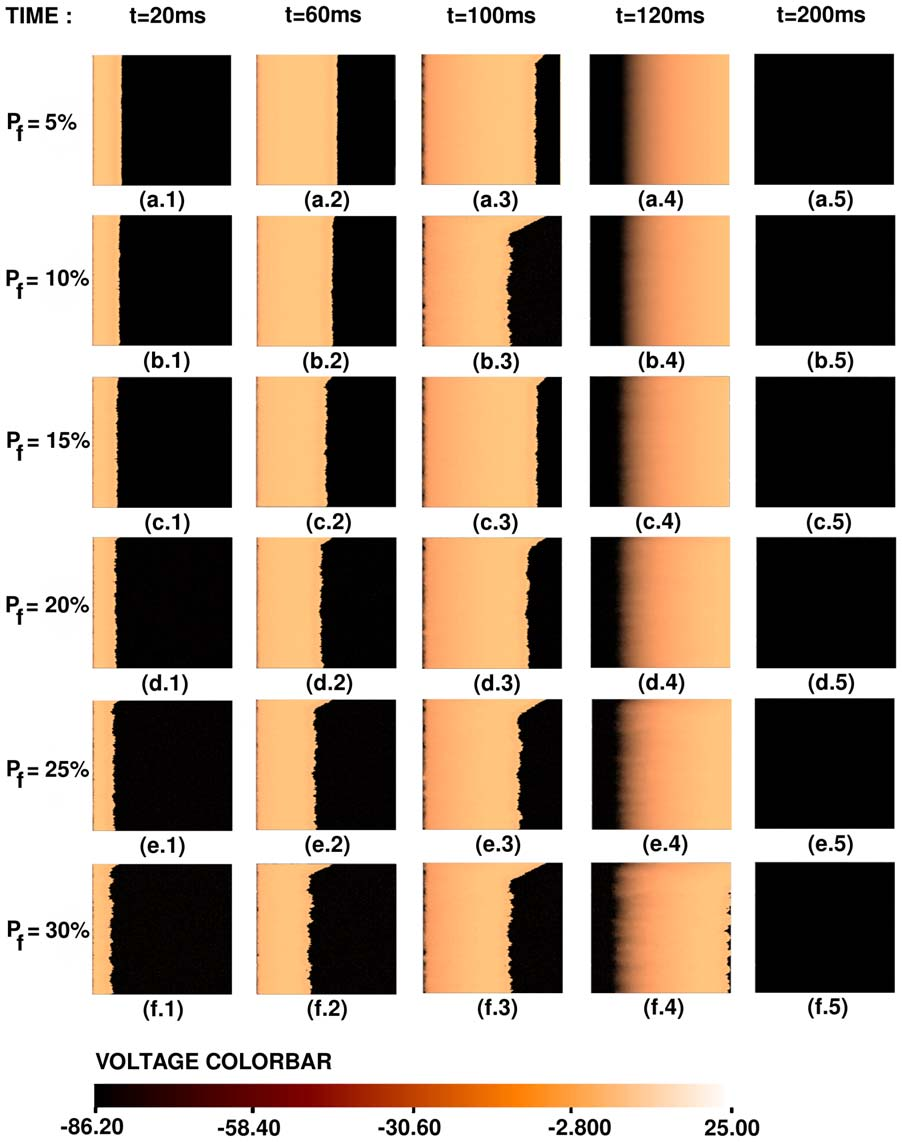
Pseudocolor plots of the local membrane potential 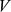 illustrating plane-wave propagation through a mural slice of our 2D simulation domain with a random distribution of myocytes and fibroblasts. Here we consider the moderate-coupling case 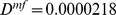 cm^2^/ms, and 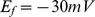 and in (a.1)–(a.5) the percentage of fibroblasts 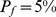, in (b.1)–(b.5) 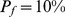, in (c.1)–(c.5) 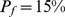, in (d.1)–(d.5) 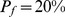, in (e.1)–(e.5) 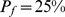, and in (f.1)–(f.5) 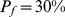. (For full spatiotemporal evolutions see Video S1.).

Representative pseudocolor plots of the local membrane potential 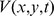 are given in Fig. 7 for several values of the time 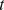 to illustrate plane-wave propagation, through a 2D mural slice, in the moderate-coupling case, for different values of 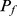. (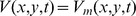, if the site 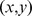 is occupied by a myocyte, and 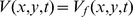, if the site 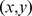 is occupied by a fibroblast.) We do not see behaviors of types **R2–R5** here; the plane wave propagates through the medium with a slightly corrugated wave front (region **R1**). Video S1 shows the spatiotemporal evolution of the plane waves in Figs. 7 (a.1), (b.1), (c.1), (d.1), (e.1), and (f.1).

**Figure 8:**
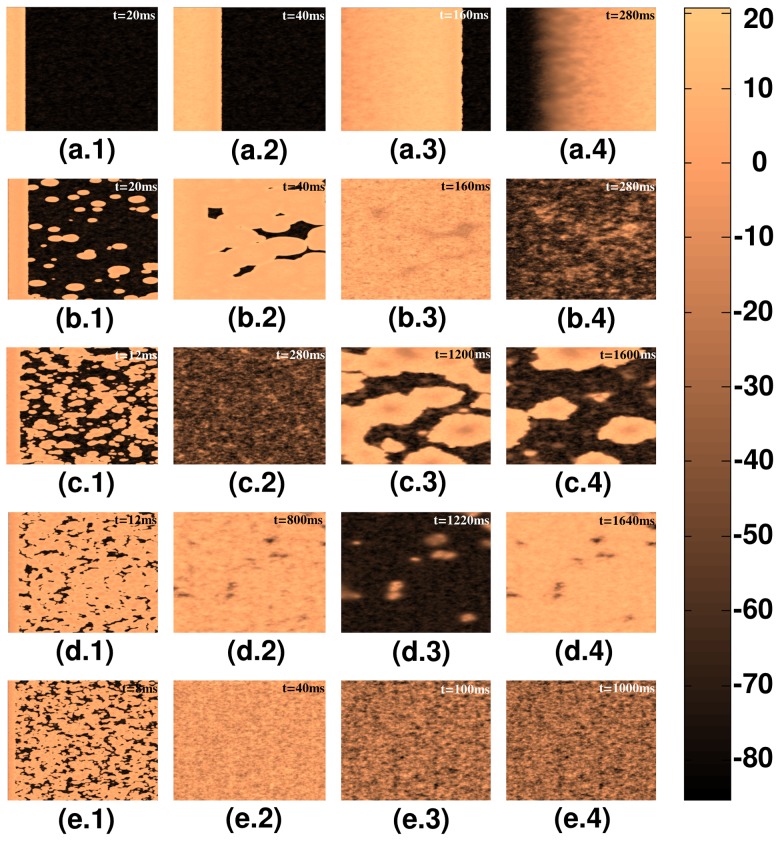
Pseudocolor plots of the local membrane potential 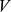 illustrating plane-wave propagation through a mural slice of our 2D simulation domain with a random distribution of myocytes and fibroblasts. Here we consider the strong-coupling case and different régimes in the stability diagram of Fig. 6; (a.1)–(a.4) propagation in régime **R1** (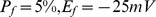); (b.1)–(b.4) propagation in régime **R2** (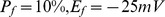); (c.1)–(c.4) propagation in régime **R3** (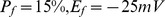); (d.1)–(d.4) propagation in régime **R4** (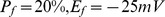); (e.1)–(e.4) propagation in régime **R5** (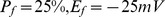).(For full spatiotemporal evolutions see Video S2.).

Analogous plots, for the strong-coupling case, of plane-wave propagation, through a 2D mural slice, are shown in Fig. 8; plane-wave propagation for régime **R1** is illustrated in Figs. 8 (a.1)–(a.4), for régime **R2** in Figs. 8(b.1)–(b.4), for régime **R3** in Figs. 8(c.1)–(c.4), for régime **R4** in Figs. 8 (d.1)–(d,4), and for régime **R5** in Figs. 8(e.1)–(e.5). The spatiotemporal evolution of these plane waves is given in Video S2.

**Figure 9:**
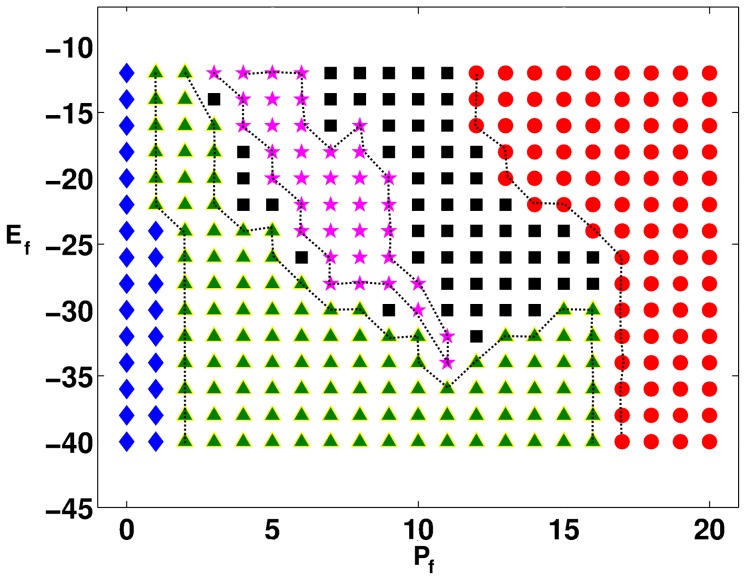
Pseudocolor plots of the local membrane potential 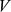 illustrating plane-wave propagation through a transmural slice of our 2D simulation domain with a random distribution of myocytes and fibroblasts. Here we consider the moderate-coupling case 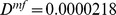 cm^2^/ms, and 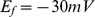 and in (a.1)–(a.5) the percentage of fibroblasts 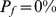, in (b.1)–(b.5) 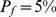, in (c.1)–(c.5) 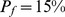, in (d.1)–(d.5) 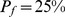, in (e.1)–(e.5) 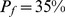, and in (f.1)–(f.5) 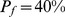. As 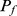 increases, not only does the distortion of the wavefront increase but the wave also propagates preferentially through the zone that has epicardial myocytes (rather than the zones with mid-myocardial and endocardial myocytes).(For full spatiotemporal evolutions see Video S3.).

**Figure 10:**
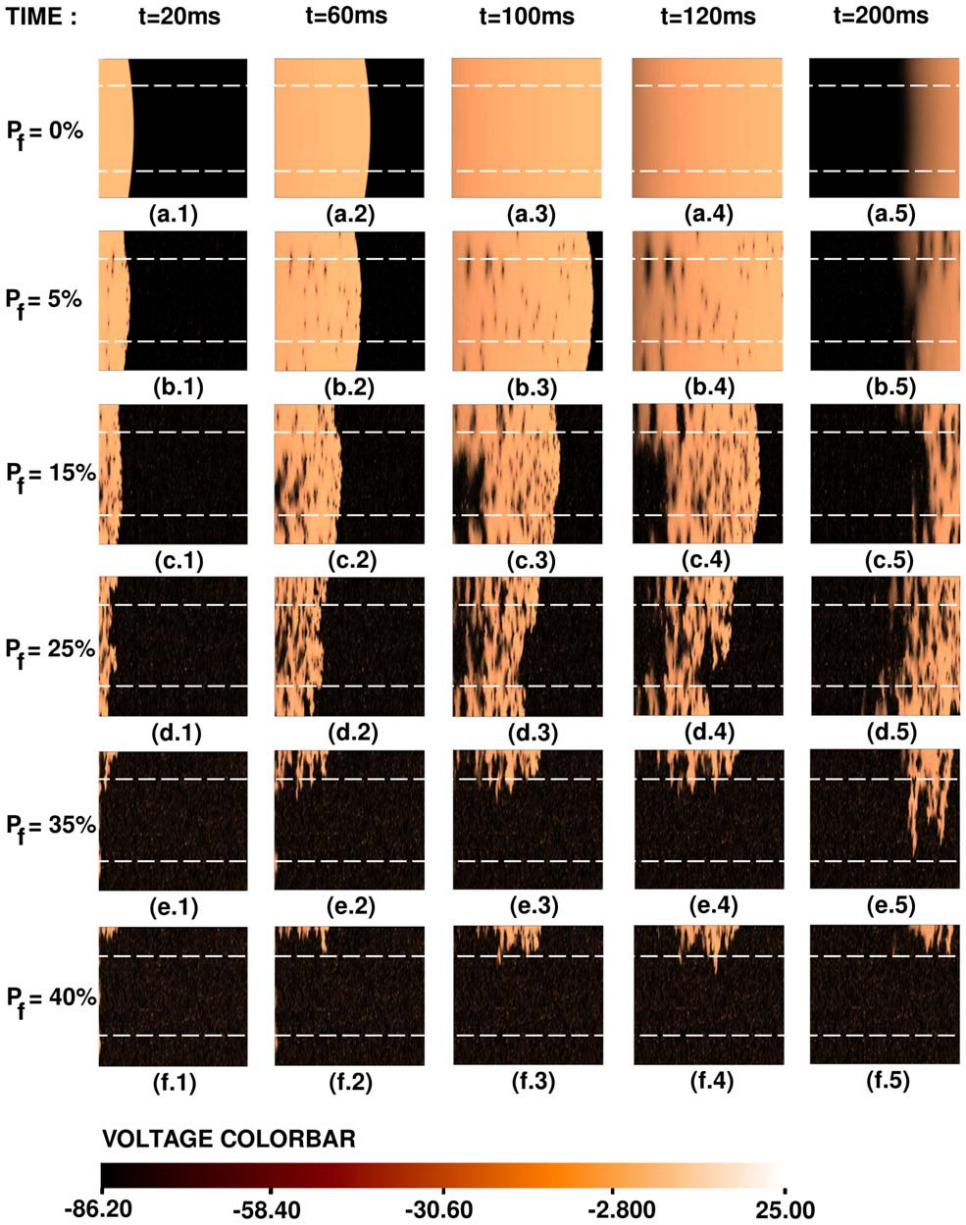
Stability diagram in the 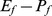 plane for plane-wave propagation through a transmural slice of our 2D simulation domain with a random distribution of myocytes and fibroblasts. The stability diagram shows the regions with spatiotemporal behaviors **R1–R5** in the strong-coupling case (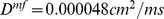); the regions **R1**, **R2**, **R3**, **R4**, and **R5** are denoted, respectively, by blue diamonds, green triangles, pink pentagrams, black squares, and red circles; the spatiotemporal evolution of plane waves in these regions is described in the text.

We turn now to illustrative studies of plane-wave propagation through a 2D transmural slice. Here too, we find the five qualitatively different spatiotemporal behaviors **R1–R5**. In the régime **R1**, which occurs both in moderate- and strong-coupling cases, the plane wave propagates smoothly through the simulation domain but with remarkable distortion. In the moderate-coupling case, at low values of 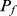, the wavefront acquires a smoother appearance than in the 2D mural slice; the smoothness begins to disappear as 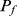 increases. Furthermore, these waves propagate differently within the three layers of the heart wall, inside the simulation domain. For sufficiently large values of 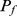, electrical conduction is partially blocked in the mid-myocardium and completely blocked in the endocardium; the excitation then travels only along the epicardium as illustrated in Fig. 9. In the strong-coupling case, régime **R1** occurs only at low values of 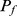, as in the moderate-coupling case; and here the wave has a smooth wave front. Régimes **R2**, **R3**, **R4** and **R5**, analogous to those in the strong-coupling case of the 2D mural slice, are also observed in the strong-coupling case of the 2D transmural slice. However, these are absent in the moderate-coupling case, in the parameter régimes that we have explored. The stability diagram, which shows the regions with spatiotemporal behaviors **R1–R5** in the strong-coupling case, is given in Fig. 10; regions **R1**, **R2**, **R3**, **R4**, and **R5** are denoted, respectively, by blue diamonds, green triangles, pink pentagrams, black squares, and red circles. Representative pseudocolor plots of the local membrane potential 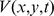 are given in Fig. 9 for several values of the time 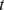 to illustrate plane-wave propagation, through a 2D transmural slice, in the moderate-coupling case, for different values of 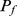. We do not see behaviors of types **R2–R5** here; the plane wave propagates, through the medium, with a distorted wave front (region **R1**). Video S3 shows the spatiotemporal evolution of the plane waves in Figs. 9 (a.1), (b.1), (c.1), (d.1), (e.1), and (f.1).

**Figure 11:**
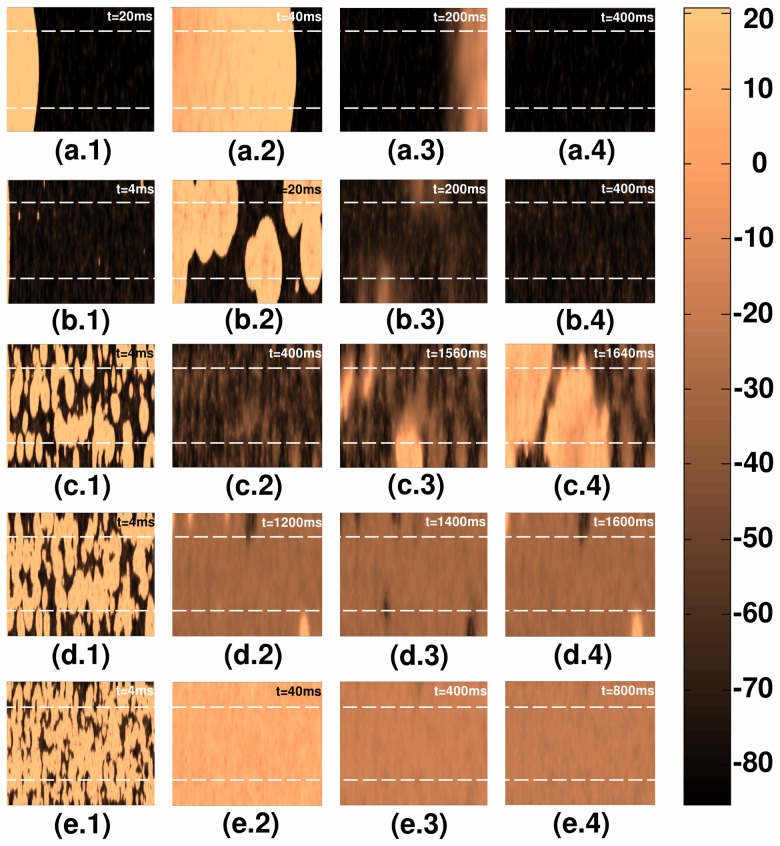
Pseudocolor plots of the local membrane potential 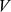 illustrating plane-wave propagation through a transmural slice of our 2D simulation domain with a random distribution of myocytes and fibroblasts. Here we consider the strong-coupling case and different régimes in the stability diagram of Fig. 10; (a.1)–(a.4) propagation in régime **R1** (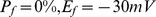); (b.1)–(b.4) propagation in régime **R2** (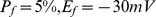); (c.1)–(c.4) propagation in régime **R3** (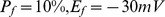); (d.1)–(d.4) propagation in régime **R4** (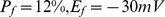); (e.1)–(e.4) propagation in régime **R5** (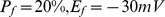). (For full spatiotemporal evolutions see Video S4.).

Analogous plots, for the strong-coupling case, of plane-wave propagation, through a 2D transmural slice, are shown in Fig. 11; plane-wave propagation for régime **R1** is illustrated in Figs. 11 (a.1)–(a.4), for régime **R2** in Figs. 11(b.1)–(b.4), for régime **R3** in Figs. 11(c.1)–(c.4), for régime **R4** in Figs. 11 (d.1)–(d,4), and for régime **R5** in Figs. 11(e.1)–(e.5). The spatiotemporal evolution of these plane waves is given in Video S4.

### Dependence of the conduction velocity and the wavelength on the percentage of fibrosis

**Figure 12:**
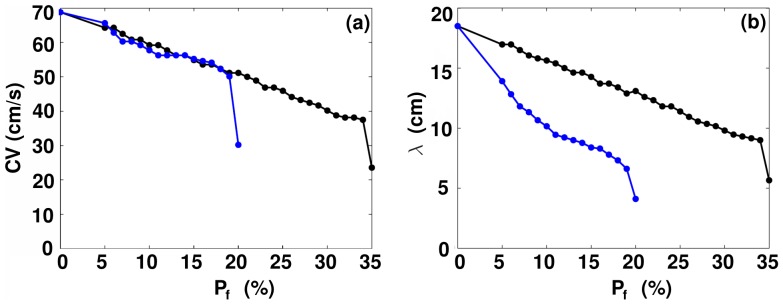
The dependence of the plane-wave conduction velocity 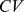 and wavelength 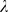 on the percentage of fibrosis 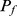. Plots of (a) 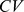 and (b) 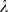 versus 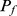 for the moderate-coupling case (solid black line with filled black circles), i.e., 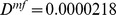 cm^2^/ms, and the strong-coupling case (solid blue line with unfilled blue circles), i.e., 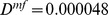 cm^2^/ms.

We characterize the influence of fibroblasts on plane-wave propagation through our mathematical model for myocardial tissue with fibroblasts by studying the dependence of the plane-wave-conduction velocity 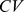 and the wavelength 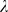 on the percentage of fibrosis 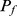; we present illustrative studies at a fixed value of the resting membrane potential of fibroblasts, namely, 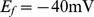; we choose this value because, from our single-MF-composite studies, it is evident that, at such a moderately low value of 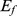, the MF composite responds to electrical stimuli as in the régime **R1**, so it is convenient to measure 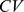. When 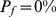 we find that 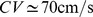, the typical value for plane-wave propagation through the human myocardium; and 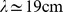. As we increase 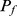, in the moderate-coupling case, 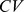 decreases gradually, as does 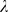. When the MF diffusive coupling is strong, 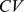 decreases gradually at first, but then, once the fibroblast clusters become large enough to generate target waves that can annihilate the plane wave, 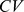 falls rapidly to zero. The medium then may or may not show conduction blockage, depending on whether it has passed into the régime **R5**, or is still in **R2**, **R3**, or **R4**. Plots of 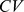 and 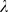 versus 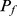 are given, respectively, in Figs. 12 (a) and (b), for both moderate-coupling (open blue circles) and strong-coupling (filled black circles) cases.

### Influence of diffuse fibrosis on spiral waves in 2D

**Figure 13:**
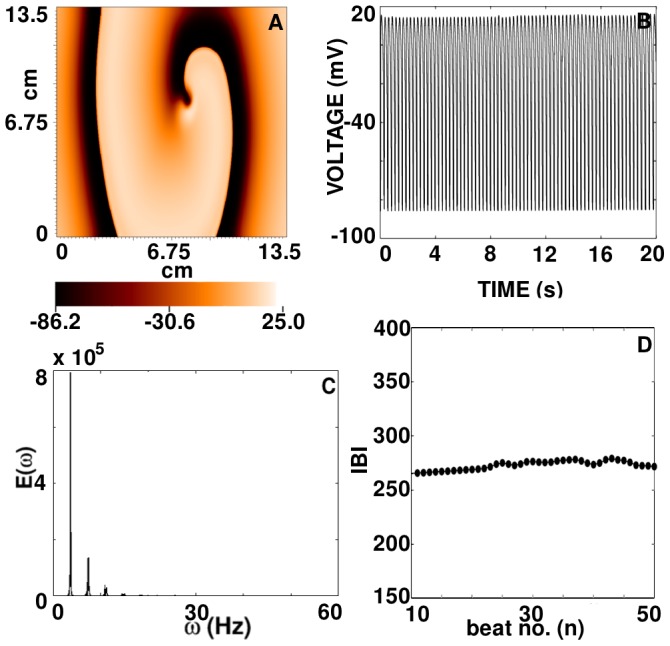
Spiral-wave dynamics in the absence of fibroblasts (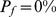) in a 2D simulation domain with myocytes of the mid-myocardial type. (A) Illustrative pseudocolor plot of the transmembrane potential 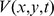 showing a spiral wave at 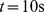. (B) A plot of the time series of 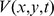 recorded from the representative point 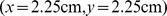; (C) a plot versus the frequency 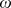 of the power spectrum 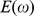 of this time series; (D) a plot of the inter-beat interval (IBI) versus the beat number 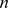 for this time series (here we have discarded the first 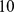 beats to remove the initial transients.

e now explore the dynamics of spiral waves of electrical activation in our mathematical model in the presence of fiber anisotropy and diffuse fibrosis. We start with a monolayer of myocytes (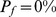) and the initial condition of Fig. 1 (a); we observe that, even after 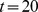 s, the medium supports only one, temporally periodic, rotating spiral wave, which shows no breaks. We call this state **SRSP** (Single-Rotating-Spiral-Periodic); a representative pseudocolor plot of the local membrane potential 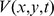, is given in Fig. 13(a) for 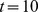 s; the time series of 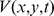, recorded from a point near the corner of the simulation domain, i.e., from (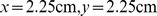), is shown alongside in Fig. 13(b); Fig. 13(c) shows the power spectrum 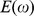 of this time series; and the corresponding plot of the inter-beat interval (IBI) versus the beat number 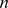 is depicted in Fig. 13(d). The simple periodicity of this time series, the appearance of a single, major peak in 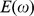 at the fundamental frequency 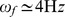, and the constancy of the IBI confirm that the spiral wave in **SRSP** evolves completely periodically in time.

**Figure 14:**
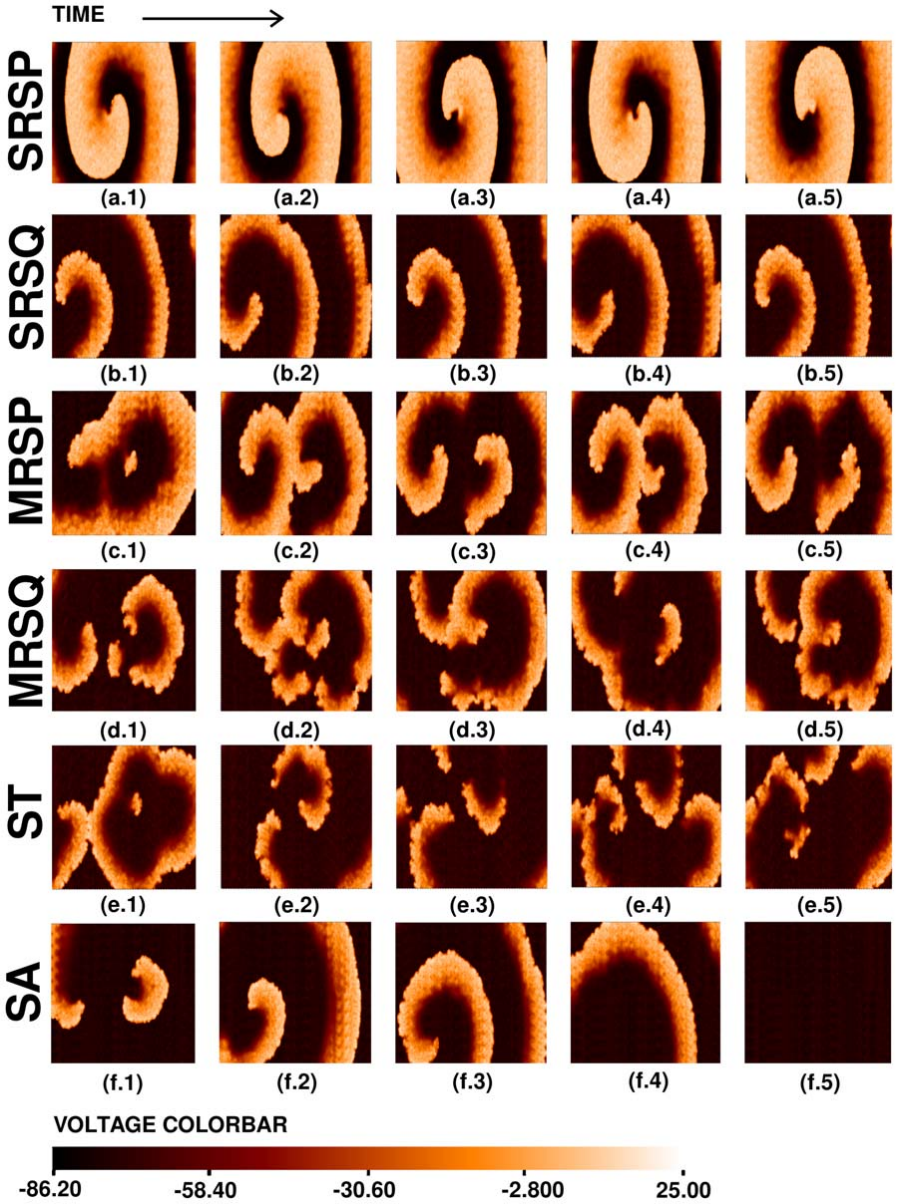
Pseudocolor plots of the local membrane potential 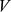 illustrating spiral-wave dynamics in a mural slice of our 2D simulation domain with a random distribution of myocytes and fibroblasts. We obtain six qualitatively different behaviors, namely, **SRSP** (Single Rotating Spiral Periodic), **SRSQ** (Single Rotating Spiral Quasiperiodic), **MRSP** (Multiple Rotating Spirals Periodic), **MRSQ** (Multiple Rotating Spirals Quasiperiodic), **ST** (Spiral Turbulence), and **SA** (Spiral Absorption). Illustrative pseudocolor plots of 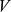 show the time evolution of a spiral wave for (a.1)–(a.5) **SRSP** with 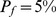, (b.1)–(b.5) **SRSQ** with 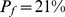, (c.1)–(c.5) **MRSP** with 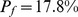, (d.1)–(d.5) **MRSQ** with 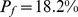, (e.1)–(e.5) **ST** with 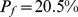, and (f.1)–(f.5) **SA** with 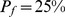. (For full spatiotemporal evolutions see Video S5.).

Next we increase 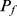 in steps of 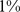. For 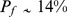, the system continues in the state **SRSP**; but, as 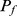 approaches 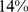, the single, completely periodic, spiral-wave develops a granular texture that increases with 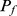; the distance from the wave-front to the wave-back also decreases. In Fig. 14 we show, for representative values of 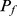, pseudocolor plots of the local transmembrane potential 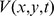; these plots illustrate the time evolution of a spiral wave in six qualitatively different states, namely, **SRSP**, **SRSQ**, **MRSP**, **MRSQ**, **ST**, and **SA**, which we have defined above; the spatiotemporal evolution of 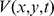 for these states is shown in Video S5. The states **SRSP** and **SRSQ** have single spirals that rotate periodically and quasiperiodically, respectively; **MRSP** and **MRSQ** have multiple spirals whose temporal evolution is periodic and quasiperiodic, respectively; the state **ST** displays spiral-wave turbulence; and in **SA** the spiral wave is absorbed at the boundaries of our simulation domain.

**Figure 15:**
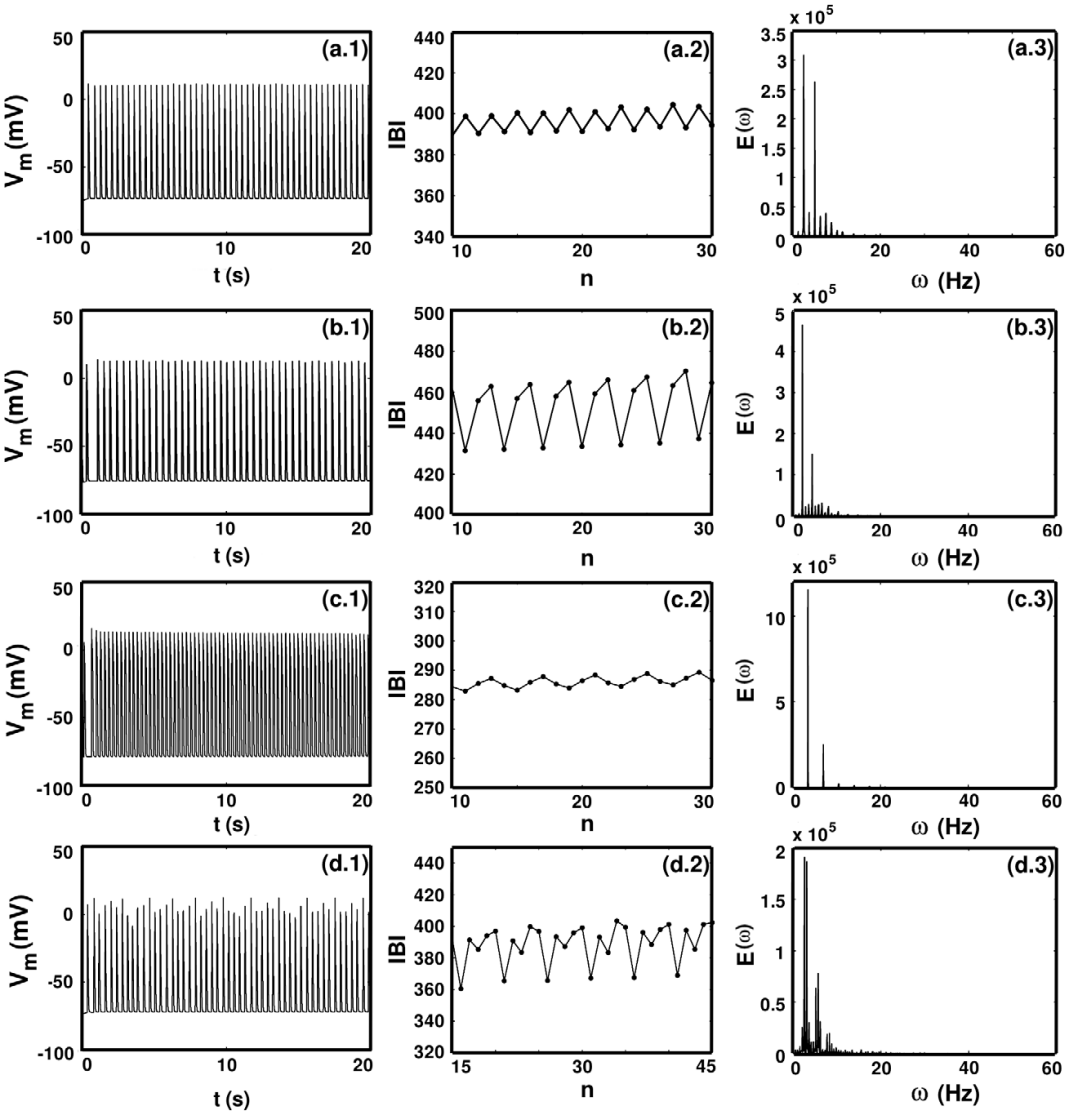
Time series of 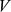 illustrating high-order temporal cycles during spiral-wave propagation in a mural slice of our 2D simulation domain with a random distribution of myocytes and fibroblasts. Plots of the time series of 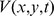, from representative points in the simulation domain, and the resulting plots of the interbeat interval IBI versus the beat number 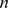 and the power spectrum 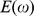 versus the frequency 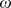 illustrating temporal 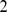-cycles (a.1)–(a.3) for 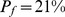, 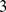-cycles (b.1)–(b.3) for 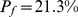), 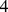-cycles (c.1)–(c.3) for 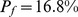), and 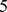-cycles (d.1)–(d.3) for 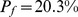; these cycles show up most clearly in the IBI plots (a.2), (b.2), (c.2), and (d.2); but their presence can also be surmised from the time series of 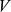 (a.1), (b.1), (c.1), and (d.1) and the sharp peaks in the power spectra (a.3), (b.3), (c.3), and (d.3)).

**Figure 16:**
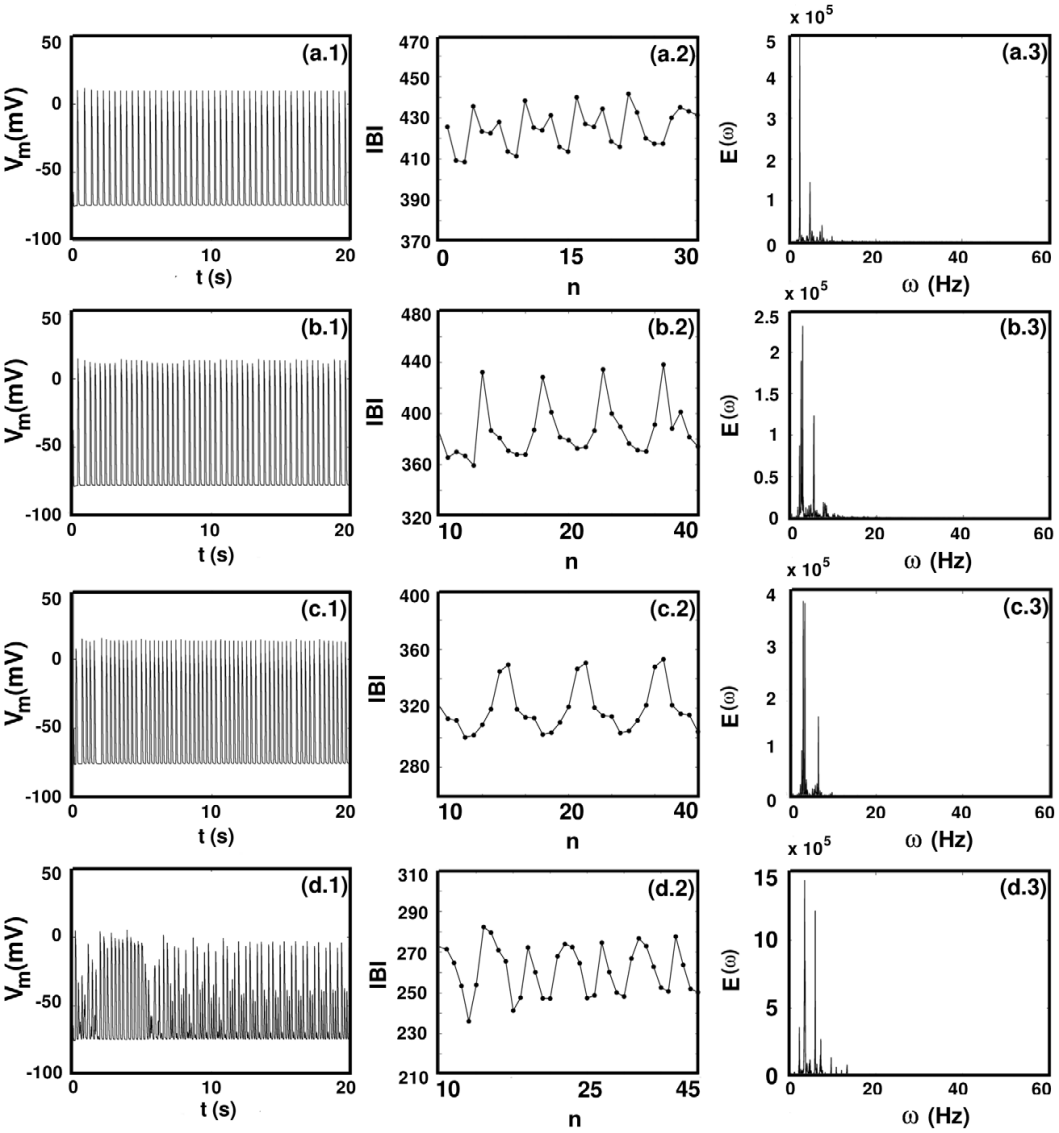
Time series of 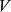 illustrating high-order temporal cycles during spiral-wave propagation in a mural slice of our 2D simulation domain with a random distribution of myocytes and fibroblasts. Plots of the time series of 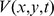, from the representative point 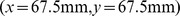, and the resulting plots of the interbeat interval IBI versus the beat number 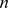 and the power spectrum 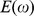 versus the frequency 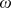 illustrating temporal 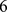-cycles (a.1)–(a.3) for 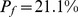, 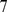-cycles (b.1)–(b.3) for 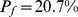), 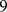-cycles (c.1)–(c.3) for 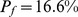), and 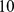-cycles (d.1)–(d.3) for 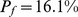; these cycles show up most clearly in the IBI plots (a.2), (b.2), (c.2), and (d.2); but their presence can also be surmised from the time series of 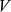 (a.1), (b.1), (c.1), and (d.1) and the sharp peaks in the power spectra (a.3), (b.3), (c.3), and (d.3)).

To examine the temporal evolution of spiral waves in these states, it is useful to look at time series of 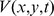, from representative points in the simulation domain, and the resulting plots of the IBI and the power spectra 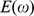. These are shown for illustrative values of 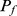 in Figs. 15 and 16.

In Figs. 15 (a.1)–(d.3) we have chosen the values of 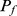 so that we can show examples of temporal 2-cycles (Figs. 15 (a.1)–(a.3) for 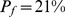), 3-cycles (Figs. 15 (b.1)–(b.3) for 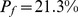), 4-cycles (Figs. 15 (c.1)–(c.3) for 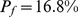), and 5-cycles (Figs. 15 (d.1)–(d.3) for 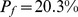); these cycles show up most clearly in the IBI plots (Figs. 15 (a.2), (b.2), (c.2), and (d.2)) but their presence can also be surmised from the time series of 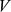 (Figs. 15 (a.1), (b.1), (c.1), and (d.1)) and the sharp peaks in the power spectra (Figs. 15 (a.3), (b.3), (c.3), and (d.3)).

In Figs. 16 (a.1)–(d.3) we have chosen the values of 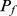 so that we can show examples of temporal 6-cycles (Figs. 16 (a.1)–(a.3) for 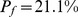), 7-cycles (Figs. 16 (b.1)–(b.3) for 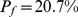), 9-cycles (Figs. 16 (c.1)–(c.3) for 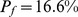), and 10-cycles (Figs. 16 (d.1)–(d.3) for 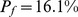); these cycles show up most clearly in the IBI plots (Figs. 16 (a.2), (b.2), (c.2), and (d.2)) but their presence can also be surmised from the time series of 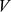 (Figs. 16 (a.1), (b.1), (c.1), and (d.1)) and the sharp, fundamental frequencies in the power spectra (Figs. 16 (a.3), (b.3), (c.3), and (d.3)).

**Figure 17:**
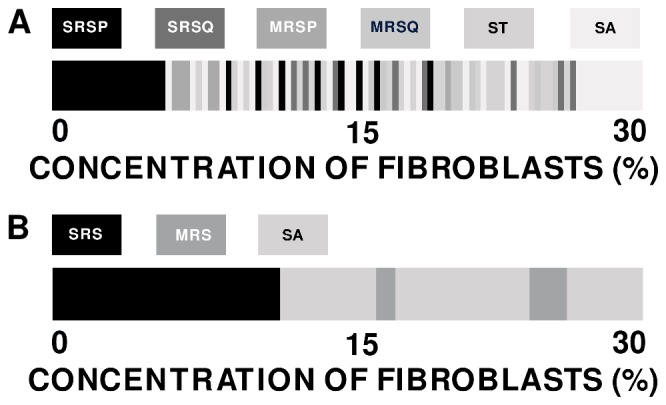
Representative band diagrams of states, in our 2D and in 3D studies, illustrating transitions between different spiral-wave (for 2D) and scroll-wave (for 3D) states as a function of 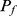. Top panel: This band diagram shows the rich sequence of transitions, from one nonequilibrium state to another, that takes us from the state **SRSP**, which occurs predominantly at low values of 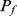, to the state **SA**, which occurs at large values of 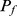; the values of 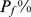 are given below the band and the six states **SRSP**- **SA** are shown by a gray scale. Bottom panel: This band diagram shows the sequence of transitions, from one nonequilibrium state to another, that takes us from the state **SRS**, which occurs predominantly at low values of 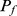, to the state **SA**, which occurs at predominantly large values of 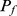; the values of 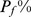 are given below the band and the three states **SRS**- **SA** are shown by a gray scale. The fine resolution of the transitions in 2D (top panel) cannot be achieved easily in 3D (bottom panel) without a prohibitive increase in computational costs.

Long time series are required to ascertain the temporal periodicity of these states. Here we obtain local time series for 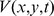, from the representative point 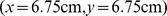, for 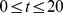 s, which corresponds to 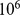 time steps; to remove the effects of initial transients, it is best to disregard data from the first 300000 iterations or so. Given plots such as those of Fig. 15 and 16, we can systematize the sequence of transitions that leads from the state **SRSP** to **SA**. For the initial conditions and the distributions of fibroblasts that we use, the sequence of transitions is shown in Fig. 17(a). The exact sequence in which these transitions occur depends sensitively on the initial conditions, boundary effects, and the realizations of fibroblast distributions within the domain, as in other nonequilibrium transitions (see, e.g., Refs. [40]–[42]).

We have found both oscillatory and autorhythmic states. Although the target waves in both these cases are similar, those in the autorhythmic case have a larger amplitude than in the oscillatory case. Note, furthermore, that the states **SRSP**, **ST**, and **SA** can be identified merely from the time series of 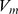, with data recorded from any representative point in the simulation domain: The time series for 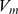, in the state **SRSP**, is completely periodic, so the plot of IBI versus the number 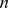 of the beat is a flat line; in the state **ST** this time series is obviously chaotic; in the state **SA** the time series is a flat line, which indicates that there is no trace of activity. In contrast, the states **SRSQ**, **MRSP**, and **MRSQ** cannot be identified unambiguously from a quick inspection of the time series of 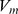 from a representative point in the simulation domain; e.g., a plot of the IBI versus 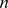 might suggest the existence of an 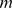 cycle, but only a careful analysis of the power spectrum 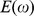 can distinguish clearly between such an 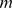 cycle and quasiperiodic temporal evolution, with more than one, incommensurate, fundamental frequencies 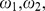 etc.; furthermore, the number of spirals or rotors cannot be identified, in these cases, unless we analyze activation movies of pseudocolor plots of 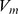 and trace the trajectories of spiral tips (these results are in consonance with earlier studies of spiral waves in mathematical models of cardiac tissue [43]–[45] without fibroblasts).

### Influence of diffuse fibrosis on scroll waves in 3D

**Figure 18:**
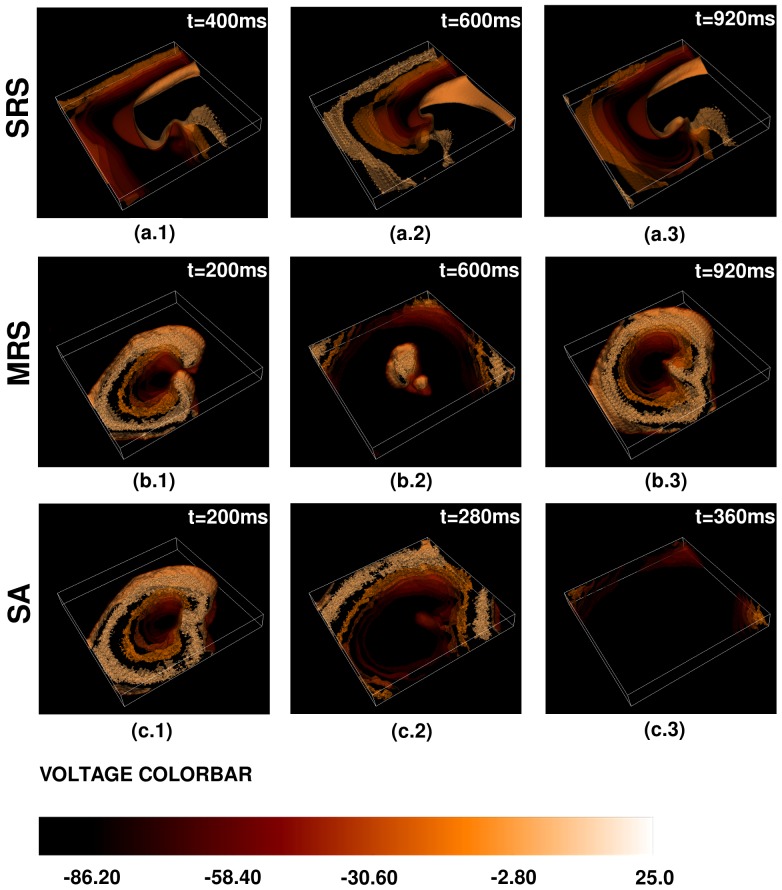
Pseudocolor isosurface plots of the local membrane potential 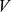 illustrating scroll-wave dynamics in a mural slice of our 3D simulation domain with a random distribution of myocytes and fibroblasts. We obtain three qualitatively different behaviors, namely, **SRS** (Single Rotating Scroll), **MRSP** (Multiple Rotating Scrolls), and **SA** (Scroll Absorption). Illustrative pseudocolor plots with isosurface slicing of 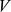 show the time evolution of a scroll wave for (a.1)–(a.3) **SRS** with 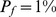, (b.1)–(b.3) **MRS** with 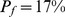, and (c.1)–(c.5) **SA** with 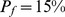. (For full spatiotemporal evolutions see Video S6.).

We consider now the dynamics of scroll waves of electrical activation in our mathematical model in the presence of fiber anisotropy and diffuse fibrosis. We start with a rectangular parallelepiped of myocytes (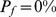) and the initial condition of Fig. 1 (b). We find that, even after 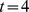 s, the medium supports only one, temporally periodic, rotating scroll wave, which does not break up further into smaller scrolls. We call this state **SRS** (Single-Rotating-Scroll). We now increase 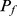 in steps of 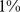 and find that, as 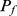 increases, this periodic, scroll-wave develops a granular texture, whose granularity increases with 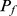; the distance from the wave-front to the wave-back also decreases. In Fig. 18 we show, for representative values of 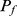, isosurface plots of the local transmembrane potential 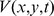 that illustrate the time evolution of a scroll wave in three qualitatively different states, namely, **SRS**, **MRS**, and **SA**, which we have defined above; the spatiotemporal evolution of 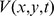 for these states is shown in Video S6. The states **SRS** and **MRS** have single and multiple scrolls, respectively; their temporal evolution may be periodic, quasiperiodic, or chaotic; to determine this unambiguously, we need far longer time series than we have been able to get with our computational resources. However, we can distinguish clearly between the states **SRS**, **MRS**, and **SA**. Given our initial conditions and the distributions of fibroblasts, the sequence of transitions in our 3D model is shown in Fig. 17(b). As we have noted in the 2D case, the exact sequence in which these transitions occur depends sensitively on the initial conditions, boundary effects, and the realizations of fibroblast distributions.

## Discussion

We have presented a comprehensive numerical study of spiral- and scroll-wave dynamics in a state-of-the-art mathematical model for human ventricular tissue with fiber rotation, transmural heterogeneity, myocytes and fibroblasts. Our mathematical model introduces fibroblasts randomly, to mimic diffuse fibrosis, in the TNNP model [16], [23] for human ventricular tissue; the passive fibroblasts in our model do not exhibit an action potential in the absence of coupling with myocytes; and we allow for a coupling between nearby myocytes and fibroblasts.

Our *in silico* study is designed to explore effectively biophysically relevant ranges of the parameters that characterize myocytes, fibroblasts, and their interactions. Thus, our work complements, in an important way, experimental studies of electrical-wave propagation in fibrotic cardiac tissue [4], [25]; and, as we have mentioned above, it extends significantly the numerical studies initiated by Panfilov [11] and ten Tusscher, *et al.* [1], [12], [13].

Simulations by Maleckar, *et al.* [46] on a rabbit ventricular model suggest that the myocyte resting potential and AP waveform, in the case of atrial arrhythmias, are modulated strongly by the properties and number of coupled fibroblasts, the degree of coupling, and the pacing frequency.

Xie, *et al.* [47] have shown that a fibroblast, coupled with a myocyte, generates a gap-junction current, which flows from the myocyte to the fibroblasts and vice versa, with two main components: an early pulse of transient outward current and a later, background current during the repolarizing phase. Depending on the relative strengths of the two components, the fibroblast-myoycte coupling can alter repolarization and 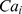 cycling alternans, at both the cellular and tissue scales. Furthermore, in a separate study [48], they show that fibroblasts affect cardiac conduction, by creating electrotonic loading and elevating the myocyte resting potential; and they suggest that fibroblast-myocyte coupling prolongs the myocyte refractory period, which may facilitate induction of reentry in cardiac tissue with fibrosis. They have used the Luo-Rudy (I) (LRI) model and a rabbit ventricular myocyte model for their studies.

Our investigation of a single MF composite, with a single myocyte coupled to 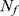 fibroblasts via a gap-junctional conductance 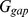, reveals five qualitatively different responses for this composite, namely, **R1–R5**. In **R1** the response of the MF composite to an external electrical stimulus is like that of an uncoupled myocyte; in **R2** this response has an additional action potential; responses **R3** and **R4** are autorhythmic and oscillatory, respectively; in **R5** the MF composite produces a single AP after which it reaches a time-independent, non-excitable state.

Our studies of 2D domains with a random distribution of fibroblasts in a myocyte background reveal that, as the percentage 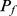 of fibroblasts increases, the 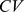 of a plane wave decreases, slowly at first and rapidly thereafter, until it reaches zero and there is conduction failure. If we consider spiral-wave dynamics in such a medium we find, in 2D, a variety of nonequilibrium states, temporally periodic (**SRSP** and **MRSP**), quasiperiodic (**SRSQ**, **MRSQ**), chaotic ( **ST**), and quiescent ( **SA**), and an intricate sequence of transitions between them (see Fig. 17 (a)). The analogous sequence of transitions for 3D scroll waves is given in Fig. 17 (b). As we have noted above, such transitions between nonequilibrium states in extended dynamical systems are known in a variety of problems including the onset of turbulence in pipe flow [40], dynamo transitions in magnetohydrodynamics [41], and the turbulence-induced melting of vortex crystals in two-dimensional soap films [42]. The precise sequence of such transitions often depends on initial conditions, boundary conditions, and, in the case we consider, on the random distribution of fibroblasts. However, the important qualitative points to note in our study are that (a) there is a variety of nonequilibrium states and (b) a rich sequence of transitions between them. These states can have important physical consequences. In particular, we speculate that the autorhythmic and oscillatory behaviors in the states **R3** and **R4** offer a possible model for ectopic foci. Thus, our studies of plane-, spiral-, and scroll-wave dynamics in our simulation domains with myocytes and fibroblasts can provide important qualitative insights into the possible effects of fibrosis on the propagation of electrical waves of activation in human ventricular tissue. In this sense, our work also builds upon the following studies: The *in-vitro* investigations of Miragoli, *et al.* [49] also suggest that fibroblasts, introduced into myocardial tissue by pressure overload or infarction, might lead to arrhythmogenesis via ectopic activity; the numerical studies of Jacquemet [50] also suggest that pacemaker-type activity can result from the coupling of cardiomyocytes with non excitable cells like fibroblasts; and Kryukov, *et al.* [51] have concluded, via *in vitro* and numerical studies of heterogeneous cardiac cell cultures and mathematical models thereof, that mixtures of excitable cells, which are initially silent, and passive cells can show transitions to states with oscillatory behavior. Interesting nonequilibrium transitions between different dynamical regimes have also been seen studied recently in a two-dimensional model for uterine tissue [52].

Our results are qualitatively in consonance with those of McDowell, *et al.* [22], who have used the Mahajan model [53] of the rabbit ventricular myocyte in a monodomain model in an anatomically realistic rabbit ventricular domain. In particular, they find that low densities of fibroblasts do not have a significant influence on the susceptibility to arrhythmias, moderate levels of fibroblasts increase the propensity for arrhythmias because of APD dispersion, and high fibroblast densities lead to conduction blockage. Their simulation domain is anatomically realistic whereas ours is not; however, we use the TNNP model for human cardiac tissue in contrast to the rabbit-ventricular model employed by them; furthermore, we carry out simulations at many more values of the fibroblast concentration than they do and, therefore, our simulations can uncover the details of the nonequilibrium transitions from single rotating spiral or scroll waves to the absorption state with no waves.

Tanaka, *et al.* [54] have studied how the random distribution of fibroblasts affects the dynamics of atrial fibrillation (AF) in sheep cardiac tissue in which heart failure (HF) has been induced artificially; they have found that the number of fibrous patches is significantly larger after HF than in a control sample. They have also carried out simulation studies by using a two-dimensional human atrial model with structural and ionic remodeling that produce HF; in these simulations they demonstrate that changes in AF activation frequency and dynamics are controlled by the interaction of electrical waves with clusters of fibrotic patches.

Muñoz, *et al.* [55] have carried out optical-mapping experiments in hetero-cellular monolayers of rat cardiac cells. Their study is designed to test whether fibroblast infiltration modifies the dynamics of spiral waves of electrical activation in such monolayers. One half of the monolayer has a randomly distributed myocyte-fibroblast mixture; the other half has a much larger concentration of myocytes (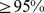) than of fibroblasts. In the former case, they find that slow (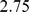 Hz), sustained re-entry is stabilized; and the wavefront propagates preferentially in the region with a high concentration of myocytes, at twice the conduction velocity (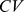) than in the region with 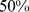 fibroblasts.

Clinically, the distribution of fibroblasts, in cardiac tissue from a normal, healthy, human heart, has been found to be of the following two types: (i) long, string-type deposits of collagen or (ii) diffuse and randomly distributed patches [56], [57]. With advancing age, structural remodeling occurs in the heart; this involves the proliferation of fibroblasts and the formation of interstitial collagen [56], [58]. It has also been established that there is a significant correlation between increased amounts of fibrotic tissue in the heart and increased incidences of atrial and ventricular tachyarrhythmias and sudden cardiac death [59]–[67]. Furthermore, the partial decoupling of muscle fibers, a decrease in 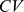, and conduction blocks have been attributed to an increase in fibrosis [57]; and there is growing consensus that impaired electrical conduction, which can lead to the formation and breakage of spiral- and scroll- waves of electrical activity, plays an important, though perhaps not exclusive, role in arrhythmogenesis.

Nguyen, *et al.* [68] have used a dynamic voltage-patch-clamp technique on adult rabbit ventricular myocytes, to reveal that the coupling of myocytes to myofibroblasts promotes the formation of early-after-depolarizations (EAD) as a result of a mismatch in early- versus late-repolarization reserve caused by a component of the gap-junction current. These cellular and ionic mechanisms may contribute to the risk of arrhythmia in fibrotic hearts.

The principal limitations of our study are that we use a monodomain description for cardiac tissue and we do not use an anatomically realistic simulation domain. These lie beyond the scope of this study. However, studies by Potse, *et al.* [69] have compared potentials resulting from normal depolarization and repolarization in a bidomain model with those of a monodomain model; these studies show that the differences between results obtained from a monodomain model and those obtained from a bidomain model are extremely small. We intend to study our MF-composite models in anatomically realistic domains and with their bidomain generalizations presently. A detailed study of diffuse fibrosis in an anatomically realistic rabbit ventricle is contained in Ref. [22]. In a separate study, we have also investigated [25] spiral-wave dynamics in a variant of our mathematical model that is motivated by the experiments of Refs. [3], [70].

Lastly, the difference between the sizes of the myocytes and fibroblasts is accounted for, in one way, in our model, namely, by virtue of the dependence of the total cellular capacitances of these two types of cells, because they depend on the surface areas of these cells. Aside from this, our model does not account explicitly for the differences in sizes between myocytes and fibroblasts. However, at large values of 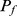, it is essential to account for fibroblast size in a more realistic way than we have. One possible way of doing this is to follow the study of Kryukov, *et al.* [51] in which 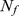 fibroblasts are allowed to couple to one myocyte; we have studied this for 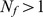 at the level of a single MF composite. The extension of this to two- and three-dimensional domains lies beyond the scope of our paper and will be taken up in a future study.

## Supporting Information

Video S1
**Plane-wave propagation in the 2D TNNP model with fiber anisotropy, randomly distributed fibroblasts, a mural section, and moderate coupling between the myocytes and the fibroblasts; panels (A), (B), (C), (D), (E), and (F), with** 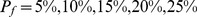 **, and** 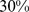 **respectively, show the spatiotemporal evolution of the plane waves in** **Figs. 7 (a.1), (b.1), (c.1), (d.1), (e.1), and (f.1)** **, via pseudocolor plots of the local transmembrane potential** 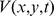 **for the time interval** 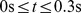 **, at 25 frames per second.**
(MPEG)

Video S2
**Plane-wave propagation, shown via pseudocolor plots of the local transmembrane potential** 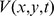 **, in the 2D TNNP model with fiber anisotropy, randomly distributed fibroblasts, a mural section, and strong coupling between the myocytes and the fibroblasts for (A) régime R1 (parameters as in** **Figs. 8 (a.1)–(a.4))** **, (B) régime R2 (parameters as in** **Figs. 8 (b.1)–(b.4))** **, (C) régime R3 (parameters as in** **Figs. 8 (c.1)–(c.4)** **), (D) régime R4 (parameters as in** **Figs. 8 (d.1)–(d,4)** **), and (E) régime R5 (parameters as in** **Figs. 8 (e.1)–(e.5)** **) for the time interval** 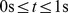 **, at 25 frames per second.**
(MPEG)

Video S3
**Plane-wave propagation in the 2D TNNP model in the presence of fiber anisotropy, transmural heterogeneity, randomly distributed fibroblasts, and moderate coupling between the myocytes and the fibroblasts.** We show the spatiotemporal evolution of the plane waves, via pseudocolor plots of the local transmembrane potential 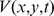, for (A) 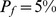 (parameters as in Figs. 9 (b.1)), (B) 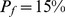 (parameters as in Figs. 9 (c.1)), (C) 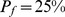 (parameters as in Figs. 9 (d.1)), (D) 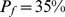 (parameters as in Figs. 9 (e.1)), and (E) 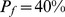 (parameters as in Figs. 9 (f.1)). The time interval covered is 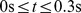, and number of frames per second is 25.(MPEG)

Video S4
**Plane-wave propagation in the 2D TNNP model in the presence of fiber anisotropy, transmural heterogeneity, randomly distributed fibroblast and strong coupling between the myocytes and the fibroblasts: We show the spatiotemporal evolution of the plane waves, via pseudocolor plots of the local transmembrane potential** 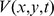 **, for (A) régime R1 (parameters as in** **Figs. 11 (a.1)–(a.4)** **), (B) régime R2 (parameters as in** **Figs. 11 (b.1)–(b.4)** **), (C) régime R3 (parameters as in** **Figs. 11 (c.1)–(c.4)** **), (D) régime R4 (parameters as in** **Figs. 11 (d.1)–(d.4)** **), and (E) régime R5 (parameters as in** **Figs. 11 (e.1)–(e.4)** **).** The time interval covered is 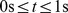, and number of frames per second is 25.(MPEG)

Video S5
**Spiral-wave dynamics in the 2D TNNP model with diffuse fibrosis.** Here we show the spatiotemporal evolution of the spiral waves in Fig. 14, for the representative values of 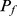 considered there, via pseudocolor plots of the local transmembrane potential 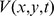 in the following six states: (A) a single spiral that rotates periodically **SRSP**, (B) a single spiral that rotates quasiperiodically **SRSQ**, (C) multiple spirals whose temporal evolution is periodic **MRSP**, (D) multiple spirals whose temporal evolution is quasiperiodic **MRSQ**, (E) spiral-wave turbulence **ST**, and (F) a state **SA** in which the spiral wave is absorbed at the boundaries of our simulation domain. The time interval covered is 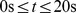, and number of frames per second is 10.(MPEG)

Video S6
**Scroll-wave dynamics in the 3D TNNP model with diffuse fibrosis: We show, via isosurface plots of the local transmembrane potential** 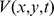 **, the time evolution of a scroll wave in the following three states (for the representative values of** 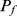 **in** **Fig. 18** **): (A) single rotating scroll SRS, (B) multiple rotating scrolls MRS, and (C) SA, which is characterized by scroll-wave absorption at the boundaries.** The time interval covered is 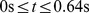, and number of frames per second is 10.(MPEG)
